# Colored Radiative Cooling: from Photonic Approaches to Fluorescent Colors and Beyond

**DOI:** 10.1002/adma.202414300

**Published:** 2025-03-04

**Authors:** Tao Wang, Ying Liu, You Dong, Xiaobo Yin, Dangyuan Lei, Jian‐Guo Dai

**Affiliations:** ^1^ Department of Civil and Environmental Engineering The Hong Kong Polytechnic University Hong Kong 999077 China; ^2^ Department of Materials Science and Engineering Department of Physics Center for Functional Photonics Hong Kong Branch of National Precious Metals Material Engineering Research Centre, and Hong Kong Institute of Clean Energy City University of Hong Kong Hong Kong 999077 China; ^3^ Department of Mechanical Engineering The University of Hong Kong Hong Kong 999077 China; ^4^ Department of Architecture and Civil Engineering City University of Hong Kong Hong Kong 999077 China

**Keywords:** color characterization, coloration, colored radiative cooling, optical materials, photonic resonant structures

## Abstract

Radiative cooling technology is gaining prominence as a sustainable solution for improving thermal comfort and reducing energy consumption associated with cooling demands. To meet diverse functional requirements such as aesthetics, switchable cooling, camouflage, and colored smart windows, color is often preferred over a white opaque appearance in the design of radiative cooling materials. Colored radiative cooling (CRC) has emerged as a prevailing technology not only for achieving a colorful appearance but also for increasing the effective solar reflectance to enhance cooling performance (through the incorporation of fluorescent materials). This paper reviews recent advancements in CRC and its profound impact on energy savings and real‐world applications. After introducing the fundamentals of CRC and color characterization, various photonic approaches are explored that leverage resonant structures to achieve coloration in radiative cooling, comparing them with conventional coloration methods based on optical materials like fluorescent pigments that can convert absorbed ultraviolet light into visible‐light emission. Furthermore, the review delves into self‐adaptive CRC materials featuring dynamic optical modulation that responds to temperature fluctuations. Lastly, the potential application of CRC materials is assessed, a comprehensive outlook on their future development is offered, and the critical challenges in practical applications are discussed.

## Graphical abstract

Colored radiative cooling (CRC) has become a prevailing technology for achieving colorful appearance and simultaneously enhancing the effective solar reflectance of cooling coatings. This review presents recent advancements in CRC and its profound impact on energy savings in real‐world applications. After introducing the fundamentals of CRC and color characterization methods, it reviews CRC coloration approaches, including photonic structural colors, complementary color absorption and Purcell‐enhanced fluorescent colors. The review concludes with self‐adaptive CRC materials featuring dynamic optical modulation and their potentially practical applications as well as critical challenges.

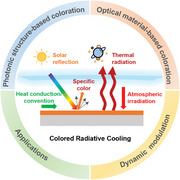

## 1 Introduction

Space cooling energy demand significantly contributes to peak electricity usage. Traditional compression‐based cooling systems result in substantial energy consumption and elevated carbon dioxide (CO_2_) emissions. Additionally, the refrigerants utilized in these systems often present risks to the ozone layer.^[^ ^1^, ^2^ ^]^ In light of the escalating global warming and the imperative of fostering a carbon‐neutral society, developing environmentally friendly, electricity‐free cooling technologies has become imperative.

Any object with a temperature above zero (0 K) can release heat through thermal radiation.^[^ ^1^ ^]^ As per the black body radiation law, the wavelength of maximum thermal emission at typical ambient temperature (around 25–30 °C) aligns with the wavelength range of the atmospheric transparency window (ATW), usually between 8 and 13 µm. This alignment enables the heat of terrestrial objects to be emitted into the frigid outer space.^[^ ^3^, ^4^ ^]^ Radiative cooling, a sustainable and passive technique, functions without additional energy input. The temperature of outdoor objects can be effectively reduced by high infrared thermal radiation transmitted through the ATW to outer space.^[^ ^5^ ^]^ Moreover, radiative cooling (RC) shows significant promise for a wide array of application scenarios, such as building energy saving,^[^ ^6^, ^7^ ^]^ personal thermal management,^[^ ^8^, ^9^, ^10^, ^11^, ^12^ ^]^ grain and chemicals storage,^[^ ^13^, ^14^ ^]^ thermal management of electronics,^[^ ^15^ ^]^ vehicles,^[^ ^16^ ^]^ space objects,^[^ ^17^ ^]^ and outdoor devices,^[^ ^18^, ^19^ ^]^ as well as the preservation of ice from melting to tackle global warming.^[^ ^20^ ^]^

Solar absorption plays a crucial role in optimizing the cooling capacity of outdoor radiative coolers. With solar irradiance level reaching ≈1000 W m^−2^, the thermal radiation power transmitted through the ATW as black body radiation is ≈100 W m^−2^ at 25 °C. Consequently, a mere 1% of solar absorption compromises at least 10% of the infrared emittance in the outdoor daytime radiative heat transfer process.^[^ ^21^, ^22^ ^]^ Traditional radiative coolers retain a white or silver appearance to ensure high solar reflectivity. However, the color properties of outdoor objects are typically prioritized to meet diverse functional and application requirements such as aesthetics, camouflage, and switchable cooling. This prioritization frequently entails selective reflection, transmission, or absorption of visible light, rather than solely focusing on total solar reflection.

Integrating color with radiative cooling serves not only aesthetics but also holds the potential to enhance cooling performance through incorporating fluorescent materials, introducing new functionalities such as switchable cooling and self‐cleaning properties, and expanding the application scope of radiative cooling across diverse domains like camouflage and agriculture. For example, fluorescence has been observed to improve effective solar reflectance in the polymer/fluorescent pigment/TiO_2_ hybrid coating, concurrently exhibiting a fluorescent color.^[^ ^23^ ^]^ Drawing inspiration from color‐switchable chameleons, color manipulation can optimize heat absorption during cold periods and enhance solar reflection during hot periods,^[^ ^24^, ^25^, ^26^, ^27^ ^]^ thereby boosting energy conservation and thermal regulation efficiency of colored radiative cooling (CRC) materials. Moreover, CRC can be tailored for visible camouflage and precise control of mid‐infrared emissivity to achieve effective thermal camouflage and radiative cooling.^[^ ^28^, ^29^ ^]^ The selective visible light transmission of CRC materials can foster vegetation growth and CO_2_ sequestration through plant photosynthesis while reflecting the remaining solar light, thereby yielding a colored cooling effect and alleviating water evaporation.^[^ ^30^, ^31^, ^32^ ^]^ Engineered hierarchical textured surfaces with low surface free energy have been designed to maintain environmentally stable optical properties. These surfaces not only confer hydrophobic self‐cleaning capabilities to the radiative cooler but also have the potential to enhance infrared radiation,^[^ ^33^ ^]^ and produce structural color through interference.^[^ ^34^, ^35^ ^]^ Therefore, developing CRC technologies is imperative to meet aesthetic preferences and required functionalities in some application scenarios.

**Figure 1:**
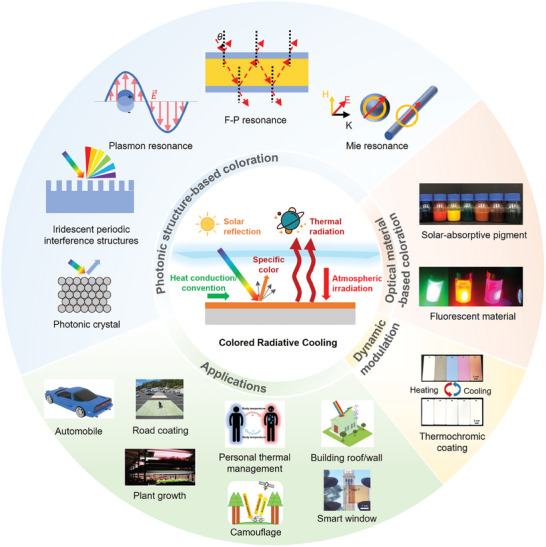
Schematic overview of the contents of this review. The overview of colored radiative cooling technologies encompasses photonic structure‐based coloration, optical material‐based coloration approaches, dynamic modulation strategies, and various applications of CRC technology. “Photonic crystal”: Reproduced with permission.^[^ ^57^ ^]^ Copyright 2024, Elsevier. “Solar‐absorptive pigments”: Reproduced with permission.^[^ ^37^ ^]^ Copyright 2023, NAS. “Fluorescent materials”: Reproduced with permission.^[^ ^67^ ^]^ Copyright 2023, ACS Publication. “Thermochromic coating”: Reproduced with permission.^[^ ^25^ ^]^ Copyright 2022, Elsevier. “Automobile”: Reproduced with permission.^[^ ^68^ ^]^ Copyright 2022. Springer Nature. “Road coating”: Reproduced with permission.^[^ ^69^ ^]^ Copyright 2020, Elsevier. “Plant growth”: Reproduced with permission.^[^ ^31^ ^]^ Copyright 2021, Springer Nature. “Personal thermal management”: Reproduced with permission.^[^ ^70^ ^]^ Copyright 2021. Springer Nature. “Camouflage”: Reproduced with permission.^[^ ^29^ ^]^ Copyright 2021, Springer Nature. “Building roof/wall”: Reproduced with permission.^[^ ^71^ ^]^ Copyright 2024. ACS Publication. “Smart window”: Reproduced with permission.^[^ ^72^ ^]^ Copyright 2018, Springer Nature.

As color is usually associated with solar absorption, coloration methodologies are crucial for achieving effective colored radiative cooling. **Figure** **1** illustrates the content of this review on CRC technologies, encompassing photonic structure‐based coloration, optical material‐based coloration, dynamic modulations and CRC applications. A diverse array of optical materials, including solar‐absorptive pigments and fluorescent pigment materials, can be seamlessly incorporated into radiative cooling systems to achieve coloration. Most colored commercial paints utilize solar‐absorptive pigments that absorb broadband solar light,^[^ ^36^, ^37^ ^]^ resulting in a notable increase in heat load. Consequently, these paints generally exhibit a solar reflectance below 0.90 and are inadequate for the sub‐ambient cooling requirement.^[^ ^6^, ^38^ ^]^ To enhance effective solar reflectance while preserving color, fluorescent pigments have been considered in CRC for their ability to convert absorbed ultraviolet light into re‐emitted visible fluorescence.^[^ ^39^, ^40^ ^]^ Aside from optical material‐based colors, researchers are also focusing on achieving the desired coloration and optimizing the spectral characteristics of photonic structures for various applications. As illustrated in Figure 1, radiative cooling using photonic structure‐based coloration employs principles such as plasmon resonance,^[^ ^41^, ^42^, ^43^, ^44^, ^45^ ^]^ Fabry‐Pérot (F‐P) resonance,^[^ ^46^, ^47^, ^48^ ^]^ Mie resonance,^[^ ^49^, ^50^, ^51^ ^]^ photonic crystals,^[^ ^52^, ^53^, ^54^, ^55^, ^56^, ^57^ ^]^ and iridescent periodic interference structures,^[^ ^35^, ^42^, ^58^ ^]^ which generate photonic structure‐induced colors while minimizing solar absorption. Additionally, thermochromic CRC has been explored to enhance adaptability to environmental temperature fluctuations and improve thermal management capabilities by modulating solar reflectivity^[^ ^25^, ^26^, ^27^, ^59^, ^60^ ^]^ or infrared emissivity.^[^ ^61^ ^]^ Various application scenarios have been realized, including automobiles, road coatings, plant growth, personal thermal management, multi‐spectral camouflage, building roofs, walls and smart windows. These applications provide customized color appearances or multiple functionalities while ensuring efficient radiative cooling performance (Figure 1, “Applications”).

To date, numerous reviews about radiative cooling (RC) materials have briefly summarized the materials/devices for CRC.^[^ ^62^, ^63^, ^64^ ^]^ In addition, several specialized reviews on CRC focus on various aspects such as the principles of radiative cooling, color calculation, and intuitive structural characteristic‐based categories of CRC materials, including patterned, multilayer, nanoparticle‐embedded, fiber‐based, and general dielectric resonant structures, among others.^[^ ^65^, ^66^ ^]^ However, to our knowledge, most reviews have not systematically summarized the photonic approaches and underlying mechanisms for CRC design, nor have they discussed strategies for balancing vibrant color with cooling performance. These aspects are crucial for developing high‐performance CRC materials and beyond. Some reviews discuss various photonic structures for RC materials but primarily emphasize enhanced solar reflectance or thermal radiation.^[^ ^62^, ^63^ ^]^ There is a need for a comprehensive review that highlights the physical mechanisms and diverse strategies for CRC materials and devices, particularly photonic approaches that tackle the challenge of coloration. Moreover, given the increasing interest in fluorescence for color design in radiative cooling, the fundamentals of CRC are incomplete without considering the fluorescence effect. Therefore, it is imperative to conduct a systematic review of CRC materials that includes the comprehensive fundamentals of CRC, coloration methodologies, dynamic spectral properties, applications, and challenges, which are also essential for the advancement of radiative cooling.

This review will elucidate the principles of CRC and the method for characterizing color, emphasizing coloration methodologies using photonic resonant structures and optical materials within CRC systems. Additionally, this review will delve into dynamic modulation design for the spectral properties in CRC and examine the application scopes of various CRC materials. Lastly, conclusions and perspectives on CRC development and applications will be provided, showcasing their versatile utility and great contributions to a sustainable human society.

## 2 Fundamentals of Colored Radiative Cooling

**Figure 2:**
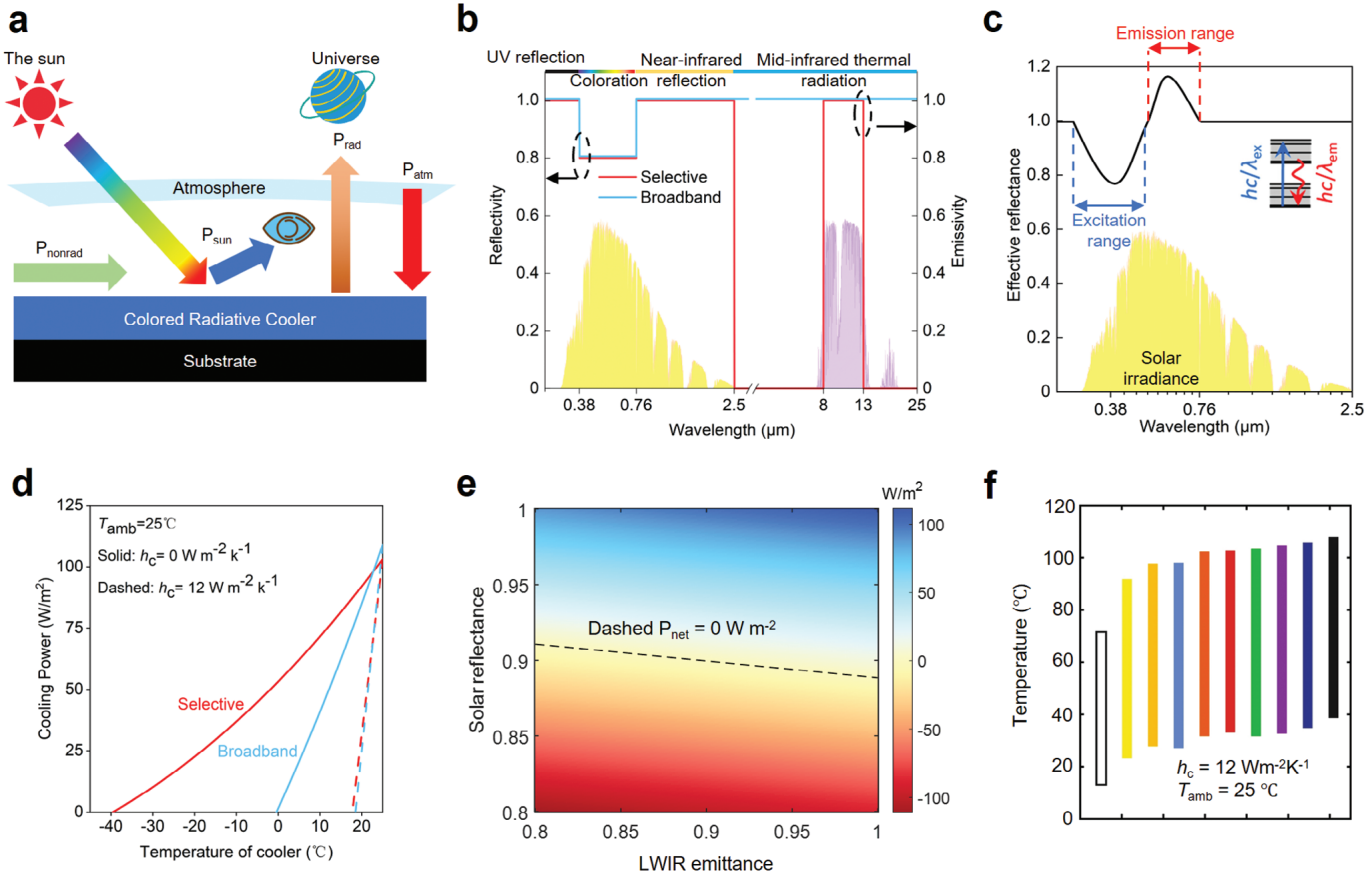
Fundamentals of Colored Radiative Cooling. a) The heat transfer process for colored daytime radiative coolers. b) Schematic of spectra properties of non‐fluorescent colored selective and broadband radiative coolers. A reflection dip represents solar absorption during coloration. Yellow shade: AM 1.5 solar irradiation spectrum. Purple shade: atmospheric transparency window. c) Schematic of effective solar reflectance of fluorescent colored radiative coolers. Reproduced with permission.^[^ ^40^ ^]^ Copyright 2022, ACS Publications. d) Cooling power variation with temperature of white radiative coolers with ideal solar reflectivity. e) Effects of solar reflectance and LWIR thermal emittance on the net cooling power of the selective radiative cooler at the initial thermal balance state when the ambient temperature is 25 °C. Solar intensity: ≈1000 W m^−2^. f) Tunable temperature range of radiative cooling photonic crystals designed by Li et al. (different colors with fixed lightness L of 60 and saturation C_ab_ of 60). Reproduced with permission.^[^ ^74^ ^]^ Copyright 2018, Springer Nature.

Thermal radiation facilitates energy transfer between objects at varying temperatures.^[^ ^3^, ^5^ ^]^ This process can be observed on Earth, where the planet emits heat into the cold outer space, resulting in a decrease in temperature during nighttime when solar irradiation is absent.^[^ ^23^, ^73^ ^]^ Notably, a colored radiative cooler can achieve outdoor daytime sub‐ambient cooling without any external energy input (**Figure** **2**a), by radiating more heat into outer space than it absorbs from sources such as the Sun, atmosphere, heat conduction, and convection with the surrounding environment. The perception of color is influenced by the visible spectra interacting with the human eye, as depicted in Figure 2a. Typically, color is influenced by the optical properties of the surface of interest and the illumination sources within visible wavelengths, including the reflectance, transmittance, absorbance, and luminescent characteristics of the object and the spectral composition of the illumination. For outdoor objects during the day, the Sun serves as the illumination source, with solar irradiation providing the spectral input.

### 2.1 Multiband Spectral Control for Colored Radiative Coolers

Colored radiative coolers typically cannot completely reflect incident visible light; otherwise, they would appear white. The design of colored radiative cooling focuses on precise spectral control across a broad spectrum, encompassing ultraviolet (UV), visible (VIS), near‐infrared (NIR), and mid‐infrared (MIR) wavelengths. Figure 2b illustrates the spectral characteristics of two categories of common non‐fluorescent colored radiative coolers with broadband and selective emissivity. Specifically, a) within the solar spectrum range of 0.25–2.5 µm, achieving zero UV and NIR absorptance (100% reflectance) is crucial to prevent solar heating. Color results from spectral‐controlled visible light reflections, which can be achieved through the establishment of photonic structures or the integration of optical materials, as detailed in the subsequent sections (“Photonic structure‐based coloration in CRC” and “Optical Material‐based Coloration in CRC”). b) In the long‐wave infrared (LWIR) transmission window spanning 8–13 µm, maintaining an emissivity of 1.0 enables efficient heat dissipation to the cold outer space through the ATW. It is important to note that atmospheric transmittance varies based on meteorological conditions; for example, high humidity results in low atmospheric transmittance and is undesired for demonstrating a cooling effect.^[^ ^75^ ^]^ Additionally, atmospheric transmittance differs among climate regions.^[^ ^73^, ^76^ ^]^ c) For the other atmosphere range in the residual MIR (2.5–8 µm and wavelengths greater than 13 µm), zero emissivity is essential to prevent excessive thermal loading from atmospheric irradiation when the cooler's temperature is sub‐ambient. Notably, in arid and subhumid areas, another apparent atmosphere transparency window in the 16–25 µm range can be harnessed for additional heat dissipation through thermal radiation.^[^ ^77^ ^]^

### 2.2 Solar Reflectance and LWIR Emittance

The design of colored radiative coolers typically integrates considerations for both solar reflectance (*R̅*_solar_) and LWIR emittance (${\bar{\varepsilon }}_{\mathrm{LWIR}}$). The non‐radiative heat load should be considered and can be calculated using Equation (11), where *h_c_* represents the nonradiative heat transfer coefficient, encompassing convection and conduction heat transfer between the cooler and its surrounding environment. In terms of the conduction heat transfer, it primarily relies on the properties of the materials surrounding the cooler, while convection heat transfer is influenced by the wind velocity passing over the cooler.^[^ ^5^, ^78^ ^]^

For the non‐fluorescent radiative coolers, the solar reflectance (*R̅*_solar_) is the ratio of the reflected solar irradiation in the *λ* of 0.25–2.5 µm to the total input of solar energy in this wavelength range.^[^ ^79^ ^]^

(1) $${\bar{R}}_{\mathrm{solar}}=\frac{\int_{0.25\mu m}^{2.5\mu m}I_{\mathrm{solar}}\left(\lambda \right)R\left(\lambda \right)d\lambda }{\int_{0.25\mu m}^{2.5\mu m}I_{\mathrm{solar}}\left(\lambda \right)d\lambda }\;\;$$

where *I*_solar_(*λ*) denotes the ASTM G173‐03 Global solar intensity spectrum, and *R*(*λ*) is the spectral reflectance of the cooler, which can be measured by commercial UV‐Vis‐NIR spectrometers.

Since the fluorescence enhances the cooling capacities through fluorescence re‐emission for compensating solar absorption, the common colored radiative coolers with fluorescent pigments would exhibit partial excitation light absorption (usually in UV and visible wavelengths), accompanied by re‐emitted fluorescence in emission wavelength ranges.^[^ ^80^, ^81^ ^]^ The effective solar reflectance (ESR), i.e., actual solar reflectance, across solar wavelength is rationally expressed as:

(2) $$\mathrm{ESR}\left(\lambda \right)=\frac{f\_b\left(\lambda \right)}{f\_i\left(\lambda \right)}=\frac{f\_bf\left(\lambda \right)+f\_bsol\left(\lambda \right)}{I_{\mathrm{solar}}\left(\lambda \right)}$$

where f_i(λ) refers to the spectral incident solar flux, and *I*_solar_(*λ*) is shown in Equation (1), f_b(λ) denotes backward energy flux within the emission wavelength range, incorporating backward fluorescence flux (f_bf(λ)) and reflected solar flux (f_bsol(λ)) at a given wavelength *λ*. For non‐emitted wavelengths, f_bf(λ)=0. As shown in Figure 2c, since re‐emitted fluorescence contributes to the backward energy flux, there exists a possibility that an over 100% reflectivity can be obtained within the fluorescence emission wavelength range according to the Equation (2).

Since the effective solar reflectance of fluorescent radiative coolers cannot be directly measured by commercial UV‐Vis‐NIR spectrometers, it can be estimated by energy flux measurements using pyranometers,^[^ ^23^ ^]^ the experimental fitting method by comparison with non‐fluorescent radiative coolers^[^ ^15^, ^82^ ^]^ and the Monte Carlo ray‐tracing method.^[^ ^31^, ^83^ ^]^

The LWIR emittance (${\bar{\varepsilon }}_{\mathrm{LWIR}}$) denotes the ratio of the thermal radiation power emitted by the cooler within the ATW, which typically spans wavelengths between 8 and 13 µm, to the integral of the thermal radiation intensity emitted by a standard blackbody within this wavelength range:

(3) $${\bar{\varepsilon }}_{\mathrm{LWIR}}=\frac{\int_{8\mu m}^{13\mu m}I_{\mathrm{bb}}\left(T,\lambda \right)\varepsilon \left(T,\lambda \right)d\lambda }{\int_{8\mu m}^{13\mu m}I_{\mathrm{bb}}\left(T,\lambda \right)d\lambda }\;\;$$

where *ε* (*T*, *λ*) represents the spectral emittance of the cooler and *I*_bb_ (*T, λ*) refers to the standard blackbody radiation intensity at a temperature of *T*, which can be calculated by Planck's law:

(4) $$I_{\mathrm{bb}}\left(T,\lambda \right)=\frac{2c^{2}\hbar }{\lambda^{5}}\frac{1}{e^{\frac{\hbar c}{\lambda k_{B}T}}-1}$$

where *k*_B_ is the Boltzmann constant, *c* is the speed of light in a vacuum environment, and ℏ is the Planck constant.

### 2.3 Cooling Power

To quantify the cooling performance of the cooler, incorporating performance indexes, i.e., the net cooling power and achievable temperature reduction compared to the ambient temperature, the thermal balance state of a daytime passive radiative cooling system has been thoroughly examined. The net cooling power of a non‐fluorescent radiative cooler with the unit area denoted as *P*_cool_, can be calculated as follows:^[^ ^3^ ^]^

(5) $$P_{\mathrm{cool}}\left(T_{c}\right)=P_{\mathrm{rad}}\left(T_{c}\right)-P_{\mathrm{atm}}\left(T_{\mathrm{amb}}\right)-P_{\mathrm{solar}}-P_{\mathrm{conv}+\mathrm{cond}}\left(T_{c},T_{\mathrm{amb}}\right)$$

where *P*_cool_ is the net cooling power, *P*_rad_, *P*_solar_, and *P*_atm_ are the thermal radiation power by the cooler, the absorbed power from solar irradiation, and the absorbed power from downwelling atmospheric radiation, respectively. *P*_conv+cond_ is the cooling power loss caused by heat convection and conduction process of the cooler with the surrounding environment. *T*_c_ and *T*_amb_ represent the temperature of cooler and ambient temperature, respectively.

As for non‐fluorescent CRC coolers, the measured solar absorbance can be used for *P*_solar_ calculation as follows:

(6) $$P_{\mathrm{solar}}=\int_{0}^{\infty }\varepsilon \left(\lambda \right)I_{\mathrm{solar}}\left(\lambda \right)d\lambda$$

where *ε* (*T*, *λ*) represents the spectral absorptance of the cooler, *I*_solar_(*λ*) is shown in Equation (1).

Regarding fluorescent radiative coolers, the effective cooling power *P*_cool, eff _(*T*_c_) should be:

(7) $$P_{\mathrm{cool},\;\mathrm{eff}}\left(T_{c}\right)=P_{\mathrm{rad}}\left(T_{c}\right)-P_{\mathrm{atm}}\left(T_{\mathrm{amb}}\right)-P_{\mathrm{solar},\;\mathrm{eff}}-P_{\mathrm{conv}+\mathrm{cond}}\left(T_{c},T_{\mathrm{amb}}\right)$$

where *P*_rad_, *P*_atm_, *P*_conv+cond_, *T*_c_, and *T*_amb_ are shown in Equation (5).

*P*_solar, eff_ denotes the effective solar heat intake, the formula is given by:

(8) $$\begin{array}{c} P_{\mathrm{solar},\;\mathrm{eff}}=\int_{0}^{\infty }f_{i}\left(\lambda \right)d\lambda -\int_{0}^{\infty }f_{b}\left(\lambda \right)d\lambda \;=\int_{0}^{\infty }\left[1-\mathrm{ESR}\left(\lambda \right)\right]I_{\mathrm{solar}}\left(\lambda \right)d\lambda \end{array}$$

wheref_i(λ), f_b(λ), and ESR(λ) are shown in Equation (2), and *I*_solar_(*λ*) is the ASTM G173‐03 Global solar intensity spectrum.

In Equations (5) and (7), *P*_rad_ has been resolved as follows:

(9) $$P_{\mathrm{rad}}\left(T_{c}\right)=2\pi \int_{0}^{\frac{\pi }{2}}sin\theta cos\theta \int_{0}^{\infty }I_{\mathrm{bb}}\left(T_{c},\lambda \right)\varepsilon \left(\lambda ,\theta \right)d\theta d\lambda$$

where *I*_bb_(*T*_c_,λ) is shown in Equation (4). ε(λ, θ) represents the emissivity of the cooler and varies with the wavelength *λ* and solid angle *θ*.

For *P*_atm_:

(10) $$P_{\mathrm{atm}}\left(T_{\mathrm{amb}}\right)=2\pi \int_{0}^{\frac{\pi }{2}}sin\theta cos\theta \int_{0}^{\infty }I_{\mathrm{bb}}\left(T_{\mathrm{amb}},\lambda \right)\varepsilon_{\mathrm{atm}}\left(\lambda ,\theta \right)\varepsilon \left(\lambda ,\theta \right)d\theta d\lambda$$

ε_atm_(λ,θ) represents the atmospheric emittance, which can be obtained with the atmospheric transmittance *t*(*λ*) given by ε_atm_ (λ,θ) =  1 − *t*(λ)^1/*cos*θ^. Here, *t*(λ) is computed by the software MODTRAN.^[^ ^84^ ^]^ where *I*_solar_ is the local solar irradiation. Separately, *P*_conv+cond_ is expressed as:

(11) $$P_{\mathrm{conv}+\mathrm{cond}}\left(T_{c},T_{\mathrm{amb}}\right)=h_{c}\left(T_{\mathrm{amb}}-T_{c}\right)$$

where *h*_c_ is the non‐radiative heat transfer coefficient caused by heat convection and conduction effect of the cooler with the surrounding environment. A empirical equation *h*_c_ = 8.3 + 2.5*v*,^[^ ^85^ ^]^ where *v* is the wind speed, can be used to approximately estimate the heat transfer coefficient in the radiative cooling system. The real‐time cooling power during the field test can be obtained through the thermal compensation method, which records the supplied power for heating the cooler to match the ambient temperature.^[^ ^86^, ^87^ ^]^

In the initial state, the net cooling power is calculated with Equation (5) when *T*_c_ = *T*_amb_. Additionally, the final‐state temperature for the cooler is computed by setting *P*_cool_ = 0 in Equation (5), when the radiative cooling system reaches a thermal balance state. To simulate the final‐state temperature in real application scenarios, real‐time data for solar irradiation intensity, average wind speed, and ambient temperature are required and used as input environmental parameters. Figure 2d demonstrates the impact of the nonradiative heat transfer coefficient *h*_c_ on the temperature reduction performance of the white selective and broadband radiative coolers with ideal reflectivity. It is apparent that nonradiative heat transfer significantly diminishes the cooling effects of the selective. For instance, at *h*_c_ = 12 W m^−2^ K^−1^, the temperature reduction effects of the selective and the broadband are very similar. However, with *h*_c_ is reduced to 0 W m^−2^ K^−1^, the selective cooler achieves a substantially lower temperature, ≈40 °C lower than the broadband cooler.

Approximately 50.2% of incident solar light energy is occupied by visible light.^[^ ^88^ ^]^ Color could bring considerable solar absorption even when the cooler exhibits high UV and NIR reflection. Regarding the effects of solar reflectance and LWIR infrared emittance on the net cooling power in the initial thermal balance state, Figure 2e illustrates that solar reflectance has a much more significant influence compared to LWIR thermal emittance. For example, a solar reflectance of ≈0.92 with an LWIR thermal emittance of 0.8 and a solar reflectance of ≈0.90 with an LWIR thermal emittance of 1.0 result in the same net cooling power of 0 W m^−2^ K^−1^. Metamerism, a concept first introduced by Li et al.,^[^ ^74^ ^]^ describes how two materials can appear identical in color while exhibiting distinct spectral properties. This concept is essential for optimizing cooling efficiency while preserving vivid colors, as it enables independent tuning of reflection/absorption in the visible and NIR wavelength range, as well as thermal emission in the mid‐infrared range. In their study, photonic crystals with the same color exhibited temperature differences exceeding 70 °C under typical outdoor conditions (Figure 2f). A more detailed design and the corresponding results are illustrated in Figure 4. This demonstrates metamerism's transformative potential for applications that demand both effective thermal management and aesthetic appeal, such as building facades, vehicles, and textiles. Notably, only the light‐yellow photonic structure achieved sub‐ambient cooling through optimized design, underscoring the importance of further exploration to advance the concept of metamerism.

### 2.4 Color Characterization

**Figure 3:**
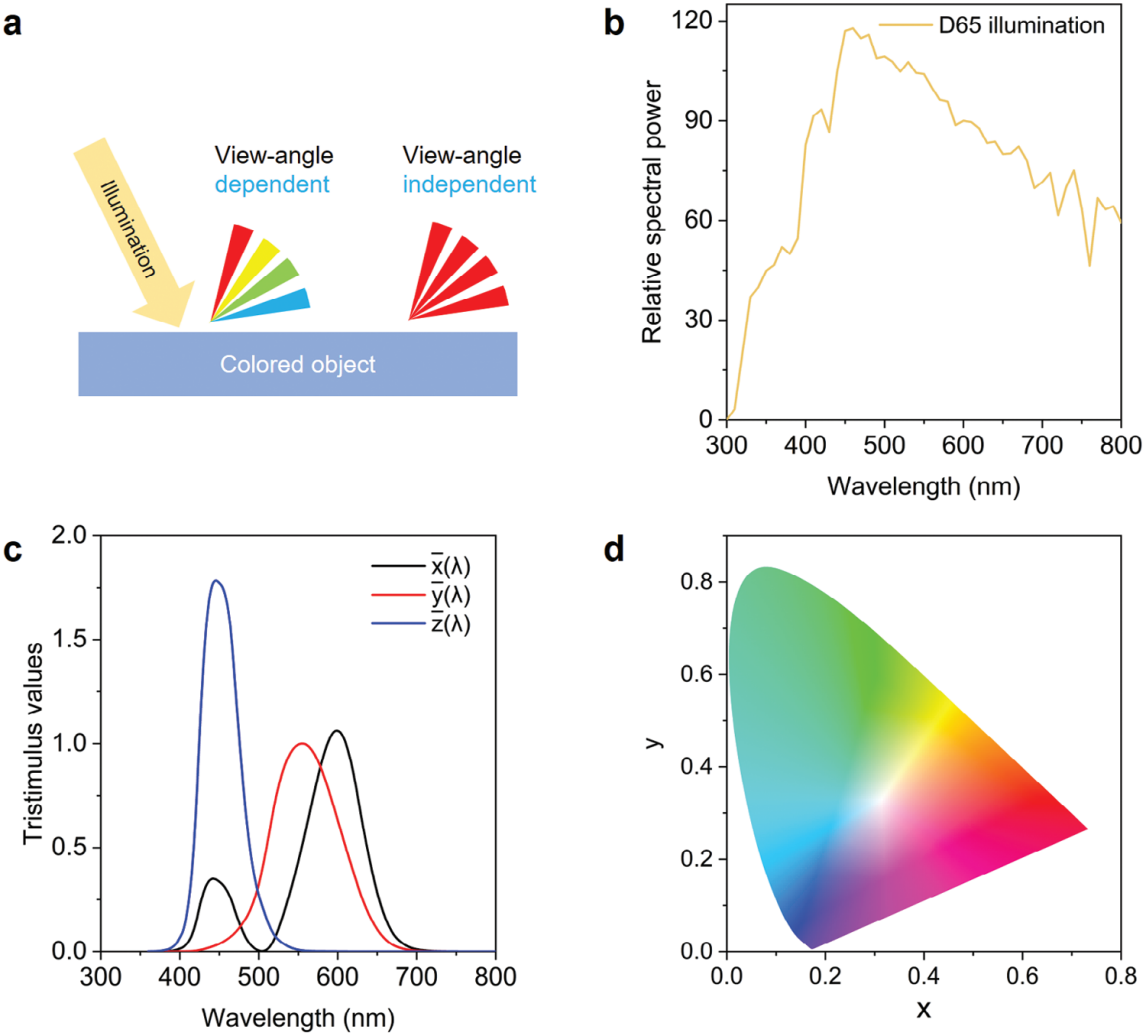
Characterization of color properties. a) Schematic of the view‐angle dependence of color. b) Standard D65 light spectrum representing typical outdoor illumination conditions. c) CIE color‐matching functions, which describe human visual sensitivity to different wavelengths. b,c) Adapted with permission.^[^ ^74^ ^]^ Copyright 2018, Springer Nature. d) CIE 1931 color space.

When characterizing and evaluating the color properties of an object, it is crucial to consider view‐angle dependence, chromaticity, lightness, and saturation. **Figure** **3**a provides a schematic illustrating the view‐angle dependence of colored objects. Structural colors generated by several interference structures exhibit view‐angle dependence and iridescence‐like effects, potentially limiting their practical applications.^[^ ^89^, ^90^ ^]^ View‐angle independent color is usually realized through selective absorption of visible light, fluorescence emission via optical materials, or the meticulous design of delicate photonic resonant structures.

The chromaticity of the measured color can be quantified via CIE coordinates. Additionally, the lightness and saturation values can be calculated to represent the lightness and vividity of the measured color, respectively. To characterize the chromaticity, according to the matching functions in the CIE XYZ system, *X*, *Y*, and *Z*, called tristimulus values, are firstly calculated to denote the level of response of human eyes to the incident light.^[^ ^91^ ^]^ Then, the spectra of Illuminant D65 (Figure 3b), which represents the standard open illumination system conditions for color characterization, is used for further calculations. The tristimulus values *X*, *Y*, and *Z* for the non‐fluorescent cooler can be obtained by the equations below:

(12) $$X=100\cdot \frac{\int I_{D65}\left(\lambda \right)R\left(\lambda \right)\bar{x}\left(\lambda \right)d\lambda }{\int I_{D65}\left(\lambda \right)\bar{y}\left(\lambda \right)d\lambda }$$

(13) $$Y=100\cdot \frac{\int I_{D65}\left(\lambda \right)R\left(\lambda \right)\bar{y}\left(\lambda \right)d\lambda }{\int I_{D65}\left(\lambda \right)\bar{y}\left(\lambda \right)d\lambda }$$

(14) $$Z=100\cdot \frac{\int I_{D65}\left(\lambda \right)R\left(\lambda \right)\bar{z}\left(\lambda \right)d\lambda }{\int I_{D65}\left(\lambda \right)\bar{y}\left(\lambda \right)d\lambda }$$

where *I*_D65_(*λ*) is spectra of Illuminant D65, $\bar{x}\left(\lambda \right)$, $\bar{y}\left(\lambda \right)\;$ and $\bar{z}\left(\lambda \right)$ are the spectra for the matching functions shown in Figure 3c, and *R*(*λ*) is the reflectance spectra of the non‐fluorescent radiative cooler. For the fluorescent radiative coolers, *R*(*λ*) in Equations (12)–(14) can be replaced by ESR(λ) as in Equation (2) for accuracy.

Finally, the chromaticity of measured color is represented by the two normalized values (*x* and *y*) calculated with Equation (15) and Equation (16), which are derived from the tristimulus values *X*, *Y*, and *Z*, and can be located in the CIE 1931 color space (Figure 3d):

(15) $$x=\frac{X}{X+Y+Z}$$

(16) $$y=\frac{Y}{X+Y+Z}$$

According to the Lab‐XYZ color space conversion,^[^ ^91^ ^]^ the lightness *L* of the measured color is calculated as below:

(17) $$L=116f\left(\frac{Y}{Y_{n}}\right)-16$$

Let *a* represent redness and greenness and *b* represent yellowness and blueness. They can be calculated as follows:

(18) $$a=500\left[f\left(\frac{X}{X_{n}}\right)-f\left(\frac{Y}{Y_{n}}\right)\right]$$

(19) $$b=200\left[f\left(\frac{Y}{Y_{n}}\right)-f\left(\frac{Z}{Z_{n}}\right)\right]$$

where *X*, *Y*, and *Z* refer to Equations ((12), (13), (14)), *X*_n_, *Y*_n_, and *Z*_n_ are the tristimulus value of the white point in the CIE XYZ color system for reference, respectively, and the function *f*(*t*) in Equations ((17), (18), (19)) can be expressed as below:

(20) $$f\left(t\right)=\left\{\begin{array}{cc} t^{\frac{1}{3}} & if\;t>{\left(\frac{6}{29}\right)}^{3}\\ \frac{t}{3\times {\left(\frac{6}{29}\right)}^{2}}+\frac{4}{29} & if\;t<{\left(\frac{6}{29}\right)}^{3} \end{array}\right.$$

Additionally, to describe the vividity of the color, the relative saturation value *C*_ab_ can be defined as:^[^ ^74^ ^]^

(21) $$C_{\mathrm{ab}}=\sqrt{a^{2}+b^{2}}$$

where *a* and *b* are shown in Equations (18) and (19), respectively.

## 3 Photonic Structure‐Based Coloration in CRC

The design of photonic structures plays a pivotal role in achieving the desired coloration and enhancing radiative cooling performance. As the Introduction section emphasizes, color typically arises from selective solar light absorption, leading to significant heat accumulation and diminished cooling efficiency. To tackle this issue, photonic structure‐based coloration is employed to attain the target color while minimizing solar absorption. Various radiative coolers that integrate structural color and high thermal radiation have been successfully developed. They can be categorized based on the photonic approaches employed for color generation, including photonic crystals,^[^ ^55^, ^74^, ^92^ ^]^ iridescent periodic interference structures,^[^ ^34^, ^93^, ^94^, ^95^ ^]^ plasmon resonant structures,^[^ ^41^, ^45^, ^96^, ^97^ ^]^ Fabry‐Pérot resonant structures,^[^ ^47^ ^]^ as well as Mie resonant structures.^[^ ^49^, ^50^ ^]^ The physical mechanisms and design strategies underlying these photonic structures in CRC are discussed in the following sections.

### 3.1 Photonic Crystals

#### 3.1.1 1D Photonic Crystals

The photonic crystal exhibits periodic variations in refractive index on the scale of the light wavelength, designed to control light propagation by creating a photonic bandgap. The bandgap alters the dispersion relation of photons. When the photonic bandgap falls within the visible light frequency range, visible light of certain wavelengths cannot propagate through the photonic crystal but forms constructive diffraction on the surface. Additionally, the light within the photonic bandgap undergoes Bragg diffraction, producing interference light that is reflected to the incident medium, resulting in structural color without view angle dependence.

**Figure 4:**
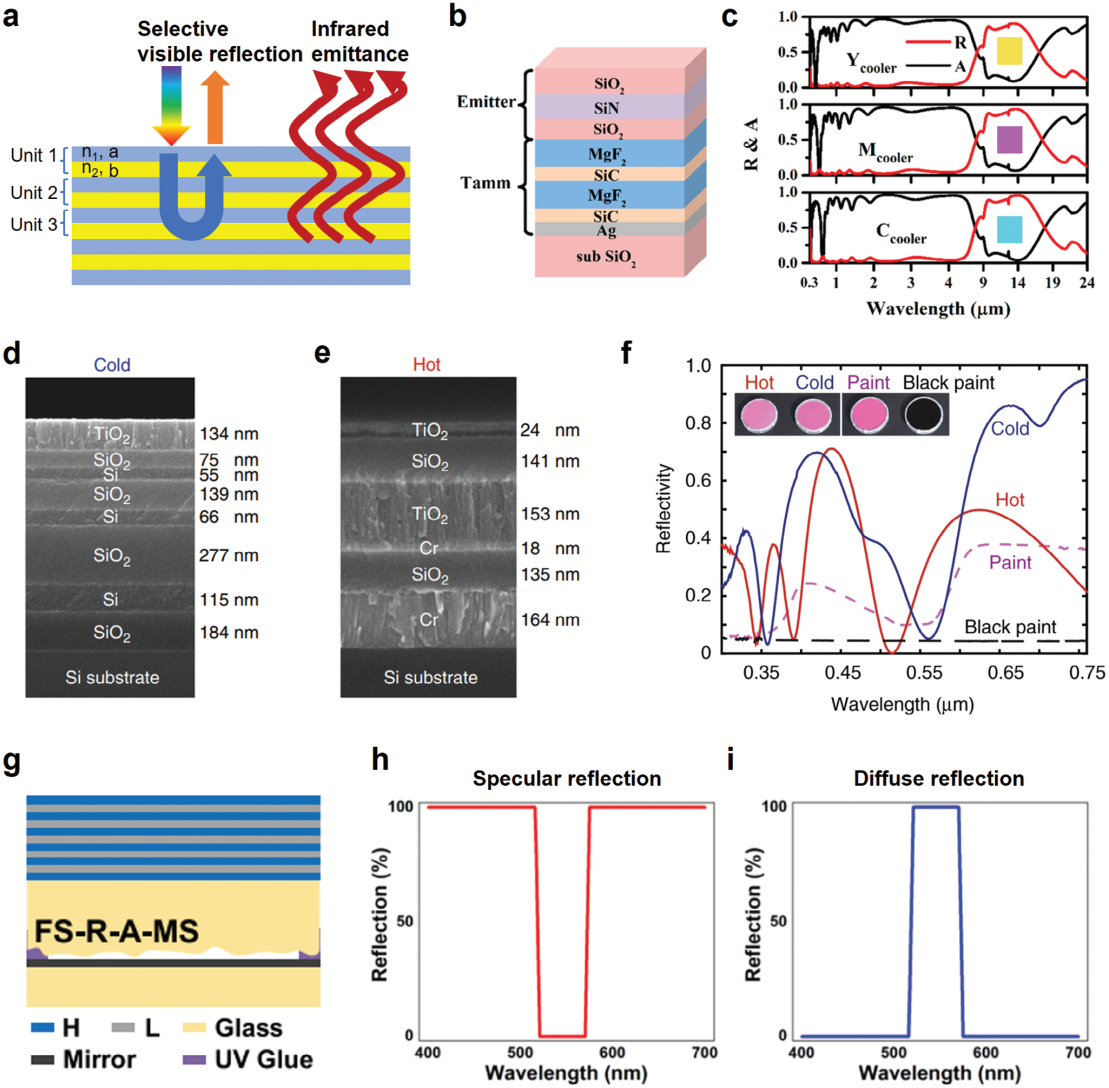
1D Photonic Crystals in CRC. a) Schematic of representation of the working mechanism of 1D photonic crystals in CRC. b) Tandem structure configuration of the designed colored radiative cooler. c) Emissivity and reflectance spectra of three coolers with different colors. b,c) Reproduced with permission.^[^ ^92^ ^]^ Copyright 2019, ACS Publications. Cross‐sectional SEM images of the d) “cold” and e) “hot” photonic structures, highlighting the material composition and layer thicknesses. f) Reflectivity spectra of the “cold” (blue curve) and “hot” (red curve) multilayer structures within the visible spectrum, illustrating metamerism. For comparison, the reflectance spectra of a commercially available pink paint (dashed purple curve) and black paint (dashed black curve) are also included. (d‐f) Reproduced with permission.^[^ ^74^ ^]^ Copyright 2018, Springer Nature. g) Structure configuration of colored cooler with nearly no solar absorption. h) Schematic of the specular reflection spectra and i) schematic of the diffuse reflection spectra of colored radiative cooler with nearly no solar absorption. g,i) Reproduced with permission.^[^ ^102^ ^]^ Copyright 2023, Springer Nature.

Photonic crystals are classified into 1D, 2D, and 3D photonic crystals based on the periodic spatial distribution of refractive index changes. 1D photonic crystals, typically implemented in multilayer structures, are primarily proposed to achieve and optimize colored radiative cooling. As shown in **Figure** **4**a, the distributed Bragg reflector (DBR) structure is often used in 1D photonic crystals for CRC. For perpendicular propagation of incident light, assuming lossless dielectrics within the operational frequency range, internal interfaces only cause reflection and transmission without absorption. The light beam will be reflected at each interface. When the incident light with wavelength *λ* propagates into the 1D photonic crystal, it undergoes reflection at the interface between the first two units, resulting in the reflected light *L*_r1_; Similarly, subsequent interfaces give rise to reflected light *L*_ri_. The difference in optical path length between the incident light and the reflected light *L*_r1_ can be expressed as:^[^ ^54^ ^]^

(22) $$d=2\left(n_{1}\times a+n_{2}\times b\right)$$

where *d* is the optical path difference, *a*, *b*, and *n*_1_, *n*_2_ denote the thicknesses and refractive indices of two dielectrics constructing 1D photonic crystal, respectively. If *d* fulfills the condition specified in Equation (22), incident light will combine with *L*_r1_, resulting in the formation of a standing wave, the remaining reflected lights *L*_ri_ will also contribute to the standing wave. Subsequently, Bragg reflection occurs, causing the incident light with wavelength *λ* to be entirely reflected. The corresponding frequency denoted as *f*  =  *c*/*λ* (*c* is the velocity of light in vacuum) can be referred to as the center of the bandgap.

(23) $$d=2\left(n_{1}\times a+n_{2}\times b\right)=m\lambda ,m=1,2,3\text{…}$$

where *a*, *b*, *n*_1_, and *n*_2_ are shown in Equation (22), *m* is a positive natural number index, and *λ* is the incident wavelength. Therefore, DBR can enhance the structure's reflectivity at a certain wavelength by optimizing its optical path length.^[^ ^98^ ^]^ For 1D photonic crystals in CRC, the desired spectral and color properties can be achieved by optimizing the constituent materials and the thickness of each layer. This allows for the reflection of the desired color, high NIR reflection, and minimized LWIR reflection using the photonic bandgap.

Sheng et al.^[^ ^92^ ^]^ utilized DBR in combination with a metallic reflective substrate and designed an alternative colored multilayer cooler based on the tandem structure. This cooler includes a tandem structure of magnesium fluoride (MgF_2_) and silicon carbide (SiC) stacking layers as a DBR on the solar reflective silver (Ag) film, along with a silicon dioxide (SiO_2_)‐sandwiched emitter composed of SiO_2_‐SiN‐SiO_2_ layers (Figure 4b). By controlling the thickness of the DBR and Ag layer, the color hues displayed on the cooler can be adjusted. The thickness of the DBR influences the color hue by adjusting the diffraction resonant wavelength, while the thickness of the Ag back reflector layer impacts color purity and lightness, particularly in comparison to subtractive primary colors (yellow, magenta, and cyan). A thicker Ag layer enhances the overall reflectivity, reduces color saturation, and increases lightness. The combined coolers exhibit high solar reflectance and broadband thermal emittance at 8–20 µm, showcasing the displayed colors (Figure 4c).^[^ ^92^ ^]^ Finally, this colored tandem cooler demonstrated a cooling power of about 52 W m^−2^, reducing the temperature of the cooler by ≈6 °C.

Bayesian optimization,^[^ ^99^ ^]^ genetic algorithm,^[^ ^100^ ^]^ and prominent memetic algorithm^[^ ^74^, ^101^ ^]^ have been used for 1D photonic crystal structure design in CRC. With the Memetic algorithm, Li et al.^[^ ^74^ ^]^ designed two multilayer structures with visually identical pink colors while displaying notable distinctions in spectral properties: one with high NIR reflection and LWIR emittance for cooling, and the other with low NIR reflection and LWIR emittance for heating. The “cold” photonic structure comprises a DBR consisting of seven alternating layers of Si and SiO_2_, with a top layer of titanium dioxide (TiO_2_) (Figure 4d, “cold”). The contrast in refractive index between silicon (Si) and SiO_2_ enables high NIR reflectance, thereby minimizing solar absorption.^[^ ^74^ ^]^ By appropriately designing the thicknesses of SiO_2_ and TiO_2_ layers, this structure primarily facilitates high thermal emission in the LWIR wavelength range.^[^ ^74^ ^]^ In contrast, the “hot” structure comprises a metal‐insulator‐metal (MIM) stacking, and three dielectric layers positioned on top of the chromium (Cr) layer (Figure 4e, “hot”). The choice of Cr over Si is attributed to Cr's intrinsic NIR absorptive properties, stemming from its high imaginary refractive index in the NIR spectrum. Additionally, Cr possesses a significantly larger imaginary refractive index than its real part in the MIR range, resulting in substantial impedance mismatch and consequently high LWIR reflectivity. Thus, this “hot” structure demonstrates high NIR absorbance with low LWIR emissivity, due to Cr's solar absorption and infrared reflection characteristics. The visible reflection spectra can be finely tuned for the desired color by carefully selecting the material properties and layer thickness. Based on the Metamerism principle, both “cold” and “hot” structures exhibit the same pink color but significantly different optical properties (Figure 4f) compared to the corresponding pink paints. Regarding cooling performance, the “cold” multilayer structure demonstrates a better temperature reduction of about 15 °C than the pink paint.

Recently, a novel photonic multilayer structure with nearly no solar absorption with colored appearance has been achieved. Figure 4g depicts this novel multilayer structure with an H‐L stacking layer atop a glass layer containing a mirror and interspace with UV glue as a spacer. FS‐R‐A‐MS denotes Film stack on Smooth surface‐Rough surface‐Air‐Mirror on Smooth surface. H refers to TiO_2_ with a high refractive index and L refers to SiO_2_ with a low refractive index. The H‐L stacking layer acts as a color filler with high emissivity within the ATW via DBR. A metallic mirror is coated onto a separate transparent substrate with a smooth surface. The two substrates are then joined together using UV glue along the edges. The diffuse reflection unit consists of a rough surface, an Ag mirror, and an air cavity. This modified configuration eliminates the effect of surface plasmon resonance on the mirror, thereby reducing unwanted solar absorption. The proposed structure exhibits a reflectivity fall in specular reflection mode (Figure 4h) while reflecting the complementary part in diffuse mode (Figure 4i) with nearly no solar absorption.^[^ ^102^ ^]^

In summary, 1D photonic crystals can achieve optimized colored radiative cooling performance through precise photonic engineering of the bandgap. It is noteworthy that 1D photonic crystals have been employed in entirely solar‐reflective radiative coolers.^[^ ^62^, ^63^ ^]^ The primary distinction between 1D photonic crystals for CRC and those for RC could lie in the selection of constituent materials, such as Si, Cr,^[^ ^74^ ^]^ and SiC,^[^ ^92^ ^]^ which exhibit lossy properties in visible wavelengths to achieve vivid coloration in 1D photonic crystal design, while the materials used in RC are lossless in the visible wavelength range to minimize solar absorption. In terms of thermal emissivity engineering, the choice of materials remains similar, utilizing SiO_2_ and TiO_2_ for their thermal radiation properties in the atmospheric window and lossless optical properties in solar wavelength. By adjusting thickness and periodicity, high NIR reflection and selective infrared emissivity can be achieved for efficient colored radiative cooling. However, their complex and costly fabrication processes, such as electron‐beam deposition and metal sputtering, limit their scalability and application. Furthermore, precise material selection, such as using Si for the “cooling” photonic crystal as depicted in Figure 4d, along with innovative structural designs, like UV glue joints for the “FS‐R‐A‐MS” photonic structure shown in Figure 4g, are essential for achieving the desired spectral properties. These requirements, however, may impede their widespread application.

#### 3.1.2 2D and 3D Photonic Crystals

**Figure 5:**
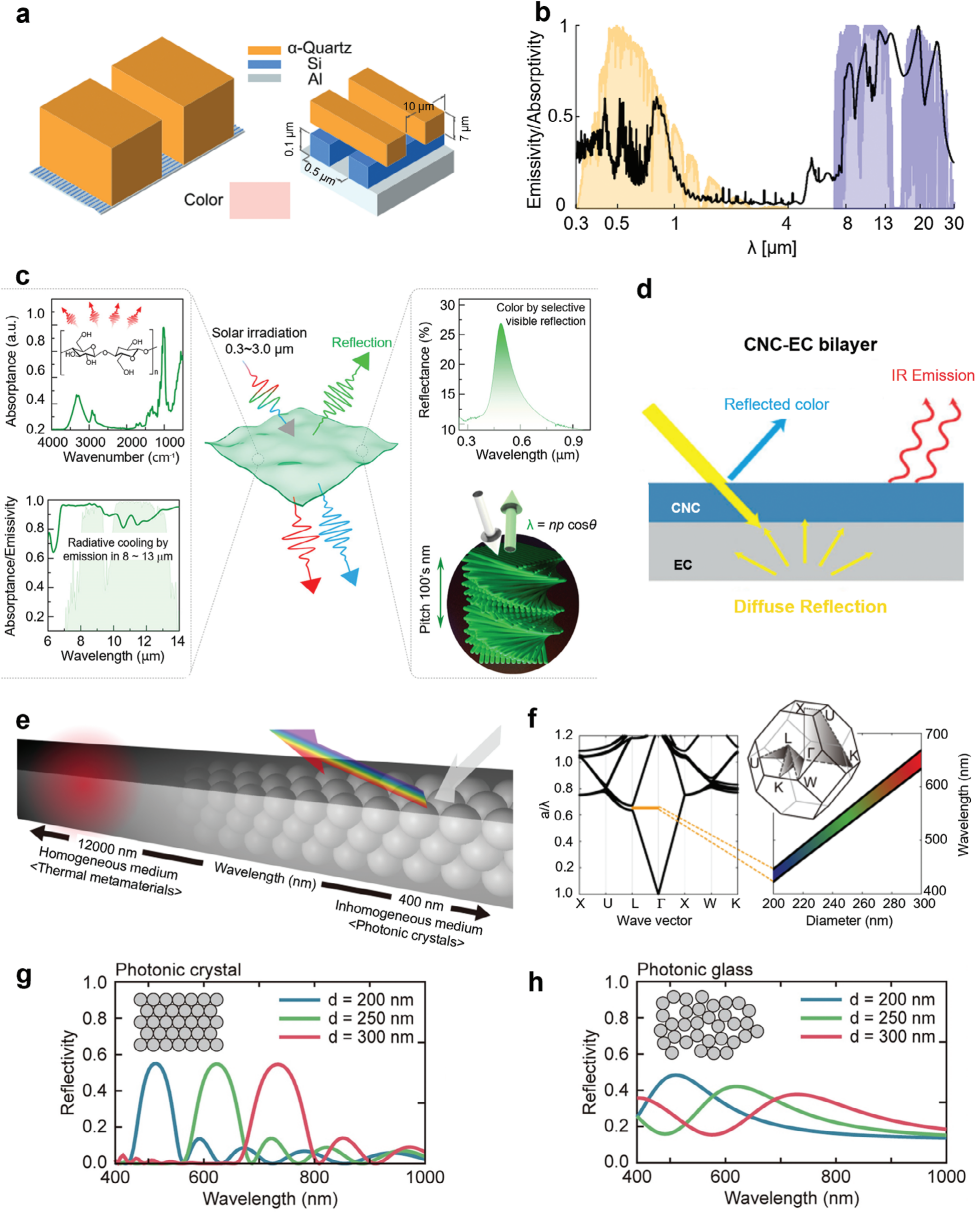
2D and 3D Photonic Crystals in CRC. a) Structure configuration of the tri‐layer colored cooler. b) Optical response of the proposed structure. a,b) Reproduced with permission.^[^ ^58^ ^]^ Copyright 2013, AIP publications. c) Structure and performance of trans‐reflective colored cellulose nanocrystal films. Reproduced with permission.^[^ ^52^ ^]^ Copyright 2022, ACS Publications. d) Structure of CNC‐EC bilayer structural colored cooling film. Reproduced with permission.^[^ ^105^ ^]^ Copyright 2022, Wiley‐VCH. e) Structure of self‐assembly colored cooler with opaline crystals. f) Band diagram for opal structure. e,f) Reproduced with permission.^[^ ^55^ ^]^ Copyright 2020, ACS Publication. Reflectivity spectra of g) photonic crystals and h) photonic glasses, both composed of silica colloids with diameters of 200, 250, and 300 nm, respectively. Reproduced with permission.^[^ ^57^ ^]^ Copyright 2024, Elsevier.

Similar to the general mechanism of 1D photonic crystals, 2D and 3D photonic crystals can reflect different colors by engineering photonic bandgaps. Zhu et al.^[^ ^58^ ^]^ introduced a daytime CRC system by implementing a bilayer periodic structure. **Figure** **5**a illustrates that the cooler consists of two functional units. The lower unit is a 2D photonic crystal. Also, the unit to reflect color consists of a periodic Si nanowires array on analuminum (Al) substrate, leveraging silicon's minimal extinction coefficient within the ATW. By adjusting the periodic spacing and length of the Si nanowires, the color appearance can be modified due to the changing constructive diffraction wavelengths of the Si nanowire array.^[^ ^58^ ^]^ The upper unit, composed of a periodic quartz bar array, which is stacked on top of the Si nanowires array, retains high solar transparency while maintaining high LWIR emittance (Figure 5b). Consequently, the entire structure achieves effective cooling without significantly affecting its color quality. This study represents the first report about the combination of radiative cooling with color appearance.

Some distinctive 3D photonic crystals produce structural color with minimal solar absorption. A representative is Cellulose Nanocrystal (CNC), which has an intrinsic chiral nematic structure. Selective light reflection can be achieved through constructive light diffraction formed in twisted layers of CNC structure. In addition, the wavelength range for photonic bandgap depends on the pitch scale of the CNC system.^[^ ^53^, ^103^, ^104^ ^]^ Since cellulose nanocrystal has a selective visible reflection, it has been applied in colored radiative cooling materials.^[^ ^52^, ^105^ ^]^ As shown in the left inset of Figure 5c, this film has a high LWIR thermal emittance due to intrinsic multiple molecular vibrations in the ATW. The chiral nematic CNC structure endows the film with selectively visible light reflection and transmission to generate color (Figure 5c, right inset). Additionally, the color appearance of the designed CNC film can be adjusted by altering the helical pitch size of the cellulose nanocrystal structure, achieved through modifications in the glucose constituent content during fabrication.^[^ ^52^ ^]^ Increasing helical pitch size leads to the redshift of the visible reflection peak due to the light diffraction law. Since the CNC film behaves a superior selective solar reflection and transmission properties, it can also be combined with a highly solar reflective bottom layer for high cooling performance. Zhu et al.^[^ ^105^ ^]^ developed a colored bilayer radiative cooling coating with a CNC layer atop a solar‐reflective Ethyl Cellulose (EC) bottom (Figure 5d). This bilayer coating exhibits a low solar absorption of ≈3% while having an over 90% infrared emittance within the ATW, demonstrating a sub‐ambient effect of ≈4 °C.

Another opal crystal structure with Bragg diffraction was utilized in fabricating a colloidal‐colored photonic crystal cooler via opal assemblies.^[^ ^55^ ^]^ As shown in Figure 5e, opal assemblies can selectively reflect visible light via the Bragg diffraction effect since the primitive cell of opal is comparable to the visible wavelength range. In the MIR wavelengths, opals function as a homogeneous medium layer, exhibiting high thermal emittance in the ATW.^[^ ^55^ ^]^ Figure 5f illustrates the band diagram for opals. By employing (111) Bragg diffraction (from the Γ to L points), the band gap of opals can be effectively opened in the visible region. This characteristic can be finely adjusted throughout the visible light wavelength range by simply modifying the size of the silica colloidal nanospheres, ranging from 200 to 300 nm. However, the opal crystal cooler faces challenges with incident angle‐dependent colorization. Recently, this limitation has been addressed by advancing colloidally paintable photonic crystals to photonic glasses, effectively resolving the angle‐dependent colorization issue. Unlike the monodisperse colloid‐based structure, photonic glasses are composed of polydisperse silica colloids. This distinction results in scattering from randomly distributed individual colloids, known as the form factor, and resonant light diffusion facilitated by the short‐range order of colloids referred to as the structural factor. As a result, photonic glasses achieve isotropic scattering and omnidirectional colorization with minimal solar absorption. Furthermore, the interplay between form and structural factors in photonic glasses enables broader spectral scattering compared to the diffractive scattering of monodisperse colloid‐based photonic crystals, as illustrated in Figure 5g,h.

In summary, 2D and 3D photonic crystals achieve coloration through diffraction effects. Specialized materials, such as chiral nematic CNCs and uniform opals, are employed to create the diffraction structure. Precise control of size parameters, like the spacing between structural units, is essential for achieving the desired color. Ultimately, the limited selection of materials and the intricate structural arrangement may impede the use of 2D and 3D photonic crystals in large‐scale CRC applications and raise durability concerns.

### 3.2 Iridescent Periodic Interference Structures

**Figure 6:**
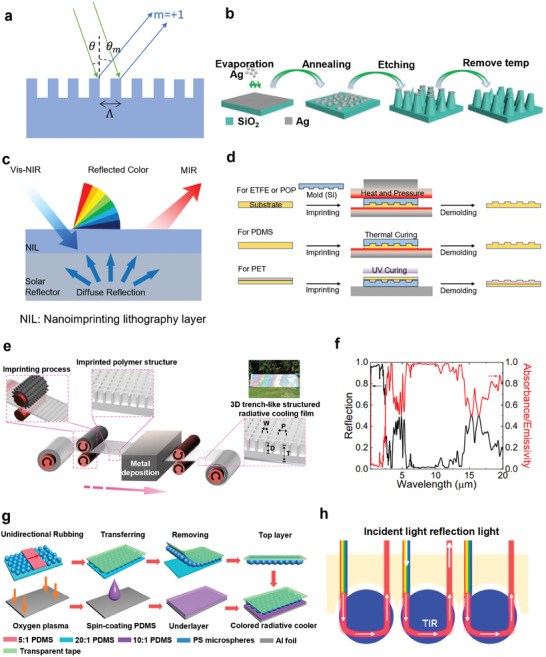
Periodic Interference Structures with Iridescent Color in CRC. a) Schematic of interference in grating structures. b) Structure of truncated SiO_2_ micro‐cones array colored cooling device. Reproduced with permission.^[^ ^107^ ^]^ Copyright 2022, Wiley‐VCH. c) Grating structure atop a solar‐reflective layer. d) Fabrication of grating structures by different polymer materials. c,d) Reproduced with permission.^[^ ^34^ ^]^ Copyright 2023, ACS publications. e) Fabrication of trench‐like structure atop a metal layer and f) spectral response of this cooler. e,f) Reproduced with permission.^[^ ^35^ ^]^ Copyright 2023, Springer Nature. g) Structure of proposed CRC with TIR‐induced color. h) Schematic for total internal reflection induced structural color. g,h) Reproduced with permission.^[^ ^93^ ^]^ Copyright 2022, ACS Publication.

Setting period size comparable to the incident light wavelength is often utilized to enhance the interaction between light and dielectric nanostructure interaction.^[^ ^106^ ^]^ The magnitude of the light‐structure interaction heavily relies on the length scales of periodic structures. Several periodic structures can display an iridescent color through interference effects. A prominent example is the diffraction grating. As shown in **Figure** **6**a, a beam of light, represented by two green rays, illuminates the binary (rectangular profile) grating. Upon interaction, the light undergoes diffraction in multiple directions, with one direction represented by the blue rays. Constructive interference occurs between the two blue rays if the difference in path lengths between adjacent green and blue rays, diffracted from identical positions on adjacent periods, equals a multiple of the light's wavelength. Constructive interference occurs when the well‐known Grating Equation (Equation 24) is satisfied.

(24) $$\sin\theta_{m}=\sin\theta +m\frac{\lambda }{\Lambda },\;m=0,\pm 1,\pm 2\cdots$$

where *m* is the diffraction order, *λ* is the incident's wavelength and *Λ* is the grating period, θ_*m*_ and θ are the angles of diffraction and incidence, respectively. The constructive and destructive interferences result in the dispersion of light into different orders of diffraction and produce an iridescent color.

Utilizing nanosized grooves with sizes ranging from 500 to 800 nm, prism gratings can generate vibrant rainbow colors. To leverage this characteristic, self‐assembly, and reactive ion etching techniques are employed to fabricate a truncated SiO_2_ micro‐cones array (Figure 6b).^[^ ^107^ ^]^ In the solar spectrum, this array forms a grating‐like structure, effectively suppressing total internal reflection between SiO_2_ micro‐cones and enhancing solar reflectivity. Additionally, the truncated SiO_2_ micro‐cones array takes advantage of a minimized refractive index mismatch at the interface, leading to enhanced infrared radiation. Through the integration of a silver reflector, the radiative cooler featuring the truncated SiO_2_ micro‐cones array showcases a wide spectrum of rainbow colors, all while maintaining an average solar reflectance of 0.95 and a remarkable LWIR emittance of 0.95.^[^ ^107^ ^]^ Consequently, this cooler can effectively reduce the temperature by 7.1 °C when exposed to direct sunlight.

Nanolithography is a cost‐effective fabrication technology widely utilized in crafting micro and nanostructures. Its integration with roll‐to‐roll fabrication techniques holds significant promise for the large‐scale production of colored radiative coolers featuring 2D periodic structures. As a common sector in optical experiments, gratings are recognized for their ability to exhibit angle‐dependent coloration akin to irradiance through interference phenomena. Zhang et al.^[^ ^34^ ^]^ designed a bilayer structure (Figure 6c) with a nanoimprinting lithography layer (NIL) atop a highly solar‐reflective bottom layer. In the design showcased in Figure 6d, a variety of polymer materials are viable options for the NIL layer, including ethylene tetrafluoroethylene (ETFE), polydimethylsiloxane (PDMS), polyolefin elastomer (POP), and polyurethane acrylate (PUA). These materials enable nanostructure fabrication via hot pressing, thermal curing or UV curing, respectively. The NIL has a narrow‐band reflection within the visible spectrum for the target color while maintaining high thermal emittance within the ATW.^[^ ^34^ ^]^ The reflective bottom layer reflects transmitted solar light from the top layer. It can be tailored according to specific requirements, such as bleached pulp sheets (∼1 mm thick) boasting an effective solar reflectance of 0.931. Figure 6e shows a 3D trench‐like nanostructures fabricated by the roll‐to‐roll method, which is atop a solar‐reflective metal layer.^[^ ^35^ ^]^ The top imprinted polymer structure endows the bilayer structure with a near‐perfect selective emissivity (Figure 6f) accompanied by irradiance‐like coloration (Figure 6e, inset). This proposed 3D trench‐like metasurface incorporates a tuning mechanism that allows for precise control over the spectral responses, thereby achieving optimal radiative cooling performance. By simulating the optical response of the structure with fixed dimensions (i.e., T = 50 µm, W = 6.5 µm, P = 8 µm) and varying parameter D ranging from 0.5 to 5 µm, the dimension parameters can be optimized for optimal performance (Figure 6e, inset). Finally, it exhibits an outstanding omnidirectional LWIR emittance of about 0.961, coupled with solar absorption of only 0.048.^[^ ^35^ ^]^ A temperature reduction of 7 °C has been achieved with an impressive cooling power of 129.8 W m^−2^.

Another colored radiative cooler achieves coloration via interferometric retroreflection.^[^ ^93^ ^]^ As shown in Figure 6g, this structure can be separated into the top and bottom parts. The top part is constituted by a transparent tap and self‐assembled polystyrene (PS) spheres. The bottom part comprises a PDMS layer and Al foil, enhancing thermal emission in MIR and solar reflection respectively. Due to the disparity in elastic moduli between components, stiff PS microspheres exhibit incomplete adhesion to the polyacrylate bonding surface of transparent adhesive tape. Multiple polyacrylate micro‐columnar structures sustain the levitation of PS microspheres above the adhesive interface, with an interstitial air layer occupying the spatial separation between them. As light transmission occurs from the higher refractive index PS microsphere medium to the lower refractive index air medium, multiple total internal reflection (TIR) events cause photon redirection along the illumination axis (Figure 6h). Upon illumination with collimated white radiation through the transparent substrate, distinct luminescent annuli become visible within individual microspheres when observed from the tape interface. This optical phenomenon arises from the dual traversal of TIR‐modified photons through the air interlayer, inducing wavelength‐dependent interference effects. Notably, chromatic characteristics generated by this retroreflective interference mechanism demonstrate tunability through controlled variation of PS microsphere dimensions. This TIR‐based CRC cooler eventually achieved sub‐ambient cooling of 4 °C.^[^ ^93^ ^]^

In short, efficient colored radiative cooling can be achieved by layering the iridescent interference structure atop a solar‐white and MIR‐black bottom layer to maximize solar reflectivity. It is important to note that some 2D periodic/patterned structures in conventional RC design are aimed at selective or enhanced thermal emission.^[^ ^63^, ^108^, ^109^ ^]^ In contrast, the resonance wavelength of 2D periodic structures for irradiation coloration falls within the visible spectrum,^[^ ^34^ ^]^ and the periodicity must be precisely tailored based on material and structural properties to achieve the desired color and spectral response in other bands. Additionally, it is feasible to achieve iridescent color and enhanced LWIR emissivity simultaneously through 3D hierarchical periodic surface structures,^[^ ^35^ ^]^ providing a method to engineer both interference colors and thermal emissivity concurrently. In addition, several periodic interference structures can be fabricated using scalable fabrication techniques such as nanolithography and the roll‐to‐roll method, which hold great potential for large‐scale applications.

### 3.3 Plasmon Resonant Structures

Plasmon resonance happens when the incident excitation wavelength's frequency matches the oscillation frequency of the free electrons in the metallic nanostructures, a highly coherent resonance occurs between the photons and electrons.^[^ ^110^ ^]^ The specific plasmon resonant wavelength depends on factors such as the sizes, shapes, and compositions of the photonic nanostructures, and the dielectric environment in which they are situated,^[^ ^106^, ^111^ ^]^ enabling the generation of different colors via selective visible light absorption.^[^ ^45^ ^]^ For spherical nanoparticles with dimensions much smaller than the resonance wavelength, the electrostatic dipole approximation can be taken, at which point the polarization rate *α* of the nanoparticle can be expressed as:^[^ ^112^ ^]^

(25) $$\alpha =4\pi a^{3}\frac{\varepsilon_{p}-\varepsilon_{m}}{\varepsilon_{p}+2\varepsilon_{m}}$$

where *a* is the nanoparticle's radius, *ε*_p_ and *ε*_m_ are dielectric constants of the nanoparticle and medium respectively. Since the electric field enhancement factor *E_loc_*/*E_in_* ∝* α*, the resonance frequency *ω*_r_ meets the relationship as follows:

(26) Reεpω=−2εm

Assumed that the dielectric constant of metallic nanoparticles can be explained by the Drude model: $\varepsilon_{p}\left(\omega \right)=1-\omega_{p}^{2}/\omega^{2}$ and *ω*_p_ is plasma frequency, *ε*_m_ is shown in Equation (25). As for spheric or ellipsoidal metallic nanoparticles, the analytic solutions of extinction and absorption efficiency can be obtained by Mie scattering theory and by considering the localized surface plasmon resonance (LSPR) effect.^[^ ^96^ ^]^ By altering the absorption efficiency spectra, narrowband visible light absorption can be obtained, which is favorable for the plasmonic color used in CRC.

**Figure 7:**
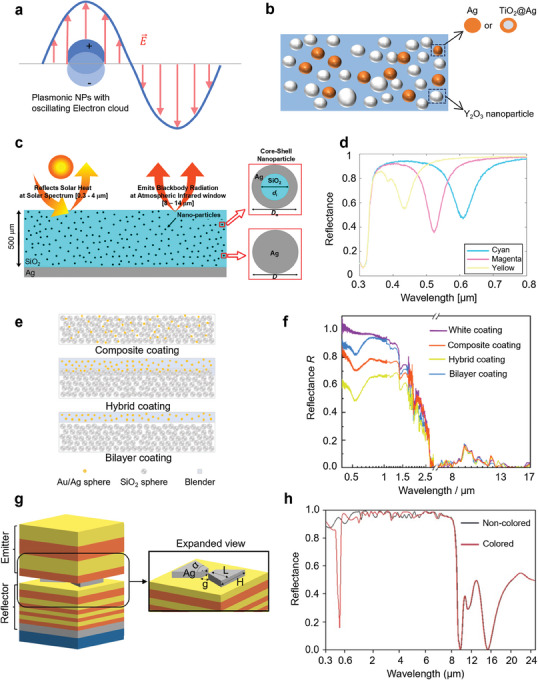
Plasmon Resonant Structures in CRC. a) Schematic of localized surface plasmon resonance. b) Structure of metallic particle embedded PMMA coating with LSPR coloration. Reproduced with permission.^[^ ^44^ ^]^ Copyright 2022, Elsevier. c) Structure of plasmonic colored radiative cooler. d) Spectra for generated subtractive primary colors via controlling the Ag shell thickness and SiO_2_ core diameter. c,d) Reproduced with permission.^[^ ^96^ ^]^ Copyright 2020, ACS Publications. e) Structure configurations of plasmonic colored coatings. f) Spectra for proposed coating structures. e,f) Reproduced with permission.^[^ ^41^ ^]^ Copyright 2022, Elsevier. g) Schematic of proposed CRC structure. The expanded view shows the nanoantenna for creating plasmonic color. h) Reflection spectra for noncolored and colored coolers that correspond to structures with and without nanoantenna, respectively. g,h) Reproduced with permission.^[^ ^45^ ^]^ Copyright 2023, Elsevier.

Metallic nanoparticles such as Ag^[^ ^44^, ^96^ ^]^ and gold (Au)^[^ ^41^ ^]^ were embedded in mid infrared‐lossy matrix material like SiO_2_^[^ ^96^ ^]^ or organic polymer materials^[^ ^41^, ^44^ ^]^ to create radiative coolers with plasmonic color and high thermal radiation. Compared to photonic crystals and iridescent periodic interference structures, particle‐embedded structures offer higher processability and are easier to fabricate on a large scale. By optimizing the size parameters of these nanoparticles, narrowband absorption of visible light in different wavelength ranges can be achieved to generate various colors while minimizing solar absorption. In addition, plasmon resonance can be used to enhance infrared emissivity by creating multi‐cavity through gap plasmon resonance^[^ ^42^ ^]^ and LSPR as depicted in **Figure** **7**a.^[^ ^43^ ^]^

Jin et al.^[^ ^44^ ^]^ proposed polymethyl methacrylate (PMMA) based colored radiative cooling coatings with Ag or TiO_2_@Ag core‐shell nanoparticles. As shown in Figure 7b, the yttrium oxide (Y_2_O_3_) particle was selected for providing efficient backscattering of solar light. By altering the volume fraction of Y_2_O_3_ nanoparticles, Ag and TiO_2_@Ag nanoparticles, composite coatings with different colors and optimized cooling performance can be modeled via Monte Carlo simulation. Leveraging LSPR, the PMMA matrix containing TiO_2_@Ag nanoparticles demonstrates serval narrowband visible light absorption peaks for the desired color and minimized solar absorption, while PMMA itself exhibits high thermal radiation in the ATW. Similarly, Yalcin et al.^[^ ^96^ ^]^ proposed a novel coating approach, as depicted in Figure 7c, involving metallic nanoparticles embedded in the SiO_2_ matrix that atop the Ag solar reflective film. The researchers discovered that SiO_2_‐based coatings incorporating plain Ag nanoparticles and SiO_2_@Ag core‐shell nanoparticles can generate subtractive primary colors, specifically yellow, magenta, and cyan, through providing required absorption peaks in the visible spectrum (Figure 7d). This effect is achieved by carefully adjusting the particle size and combining nanoparticles with different geometries. In the case of the plain Ag nanoparticle, the absorption intensity demonstrates significant particle‐size dependence, while the spectral absorption peak shift remains largely unchanged. Core‐shell architectures enable extended spectral tunability of resonance positions. All SiO_2_@Ag core‐shell nanoparticles exhibit a primary dipolar Mie resonance feature. Nevertheless, large SiO_2_@Ag core‐shell particles additionally manifest a quadrupolar resonance signature near 0.47 µm wavelength.^[^ ^96^ ^]^ This secondary narrow absorption feature at short wavelengths, which originates from the quadrupolar excitation mechanism, is less controllable than the dipole peak and produces parasitic energy absorption detrimental to cooling efficiency. Similar absorption peak locations could also be observed by using different dielectric core materials such as TiO_2_. However, it should be noted that saturation competes with the cooling performance for a specific color, where purer colors correspond to higher visible light absorption peaks.

Furthermore, the effect of different coating configurations on the optical properties of Au/Ag and SiO_2_ nanoparticle‐embedded polymer‐based coatings has been investigated.^[^ ^41^ ^]^ Figure 7e shows three proposed coating configurations: the Composite, Hybrid, and Bilayer coatings. In the Composite coating, the Au/Ag nanoparticles were blended with SiO_2_ nanoparticles to form a single‐layer coating. There are three layers in the Hybrid coating, i.e., Au/Ag particle‐based top layer, Au/Ag and SiO_2_ particle‐based middle layer, and SiO_2_ particle‐based bottom layer. As for the Bilayer coating, the topcoat and bottom layer are Au/Ag particle‐based and SiO_2_ particle‐based, respectively. These three coating configurations have margin differences in infrared emittance but different solar reflectivity while exhibiting almost the same color appearance. As shown in Figure 7f, the bilayer coating shows superiority in achieving plasmonic color while achieving better cooling performance due to its high NIR reflectance.

The LSPR photonic structure was inserted into a solar‐white multiplayer radiative cooler to constitute a CRC device. As shown in Figure 7g, a radiative cooling photonic crystal was designed first, which comprises periodic high index‐low index alternating layers (SiO_2_‐TiO_2_) on top of a thin silver layer and results in broadband reflection across the solar wavelength range. A pair of Ag nanoantennas is fabricated on the photonic crystal reflector. This bowtie nanoantenna design is evaluated for its ability in amplifying and concentrating the electric field within the gap that separates its two sections (see Figure 7g, “Expanded view”). This configuration results in a pronounced but narrowband absorption in the visible spectrum, facilitating the color manifestation of radiative cooling. Another selective emitter was put on the Ag nanoantenna structure for high LWIR thermal emittance. Although introducing this LSPR structure into a conventional radiative cooling system leads to a reduction in cooling power by ≈30%, the overall cooling power remains reasonably high at ≈60 W m^−2^. The decreased cooling power results from the diminished reflection spectrum in the visible range after incorporating a plasmonic color generator. A further benefit of the proposed model is its capacity to adjust colors by modifying the bowtie nanoantenna parameters, such as gap size, length, and height. By controlling the plasmon resonance peak, a narrow absorption peak can be achieved within the desired visible wavelength range (Figure 7h). The observed field enhancement within the gap, along with strong plasmonic coupling between the two triangular nano prisms, alter the emissivity spectrum and produce a strong yet narrow absorption spectrum.^[^ ^45^ ^]^ This mechanism allows for the generation of various colors and hues. Additionally, this bowtie nanoantenna can interact with unpolarized incident radiation in a radiative cooling system, making it suitable for cooling applications while displaying color and functioning independently of the polarization angle.

In summary, plasmonic resonance can be integrated into metallic solid or core‐shell particle configurations, facilitating straightforward coating fabrication. However, precise geometric control and the use of costly metallic materials, such as Au and Ag, may impede the large‐scale application of LSPR‐enabled CRC. In addition, for conventional radiative cooling materials, that do not consider color, plasmon resonance can be used to excite resonance within the MIR spectrum to enhance thermal radiation through metallic micro/nano structures. For example, a metal/dielectric/metal gap plasmonic structure can be used to achieve the desired spectral characteristics of thermal radiation by adjusting the width of the plasmonic cavities.^[^ ^42^ ^]^ Therefore, the selection of metallic materials and design of the micro/nano structures are need to be reconsidered and optimized when aiming at either coloration or enhancement of thermal radiation.

### 3.4 Fabry‐Pérot and Mie Resonant Structures

**Figure 8:**
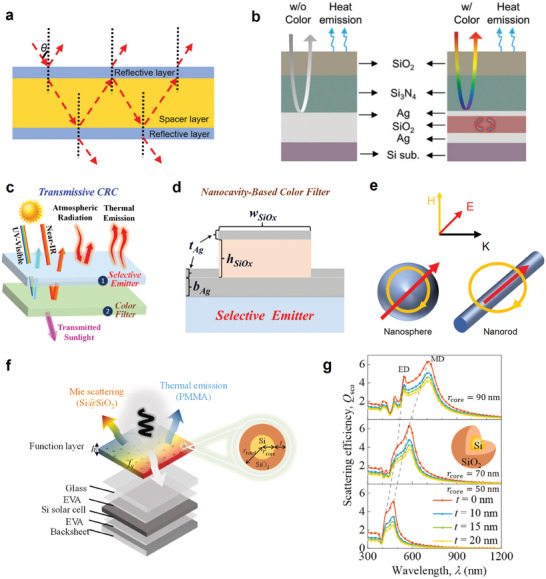
Fabry‐Pérot and Mie Resonant Structures in CRC. a) Schematic of Fabry‐Pérot resonance. b) Schematic of the colored passive radiative cooler. Reproduced with permission.^[^ ^46^ ^]^ Copyright 2018, Wiley‐VCH. c) Schematic of the Transmissive CRC. d) Structure of bottom color filter. c,d) Reproduced with permission.^[^ ^101^ ^]^ Copyright 2023, ACS publications. e) Schematic of Mie resonance. Magnetic dipole moment for sphere (left) and cylinder scattering center (right). f) Schematic of the functional layer applied for coloration of building‐integrated photovoltaic. g) Spectra for scattering efficiency with varied r_core_ and shell thickness t. f,g) Reproduced with permission.^[^ ^51^ ^]^ Copyright 2023, Elsevier.

F‐P resonance and Mie resonance are also employed in CRC material design to achieve intended coloration, alongside the attainment of superior thermal emission facilitated by the integration of thermal emitters and the incorporation of infrared‐emissive matrix materials, respectively. The F‐P cavity is an optical resonator formed by two parallel and partially reflective mirrors. As shown in **Figure** **8**a, the F‐P resonance consists of two highly reflective mirrors facing each other with a precise separation distance. The resonance condition appears when light interference positively:^[^ ^48^ ^]^

(27) $$\lambda_{m}=\frac{nL}{m},\;m=1,2,3\text{…}$$

where *λ*_*m*_ is wavelength in a vacuum, *L* is the path length, *n* is the refractive index of the media of a wave propagation, and *m* refers to the positive natural number index. The corresponding resonance frequency ω_*m*_ for index *m* is:

(28) $$\omega_{m}=\frac{c}{nL}\cdot m,\;m=1,2,3\text{…}$$

where *m, n*, and *L* are shown in Equation (27), and *c* is the light speed in vacuum. Therefore, F‐P resonance manifests resonant modes at specific wavelengths determined by the optical path length between the mirrors. An F‐P cavity can achieve structural coloration by selectively reflecting color.^[^ ^46^ ^]^ Additionally, the F‐P resonant structure can be utilized for pale‐yellow thermochromic windows^[^ ^113^ ^]^ and colored thermochromic covering for enlarged thermal emissivity modulation^[^ ^61^ ^]^ via multiple light reflections in the infrared‐lossy F‐P cavity.

A nanostructure consisting primarily of three components is depicted in Figure 8b:^[^ ^46^ ^]^ a selective emitter responsible for heat emission, a solar reflector that reflects solar light, and a MIM structure that generates subtractive primary colors, i.e., cyan, yellow, and magenta. The top part of the system features a selective emitter consisting of a SiO_2_ and silicon nitride (Si_3_N_4_) bilayer. Positioned below the emitter is an Ag solar reflective film. At the bottom layer, a MIM structure exists with an Ag‐SiO_2_‐Ag cavity, and different colors are accomplished by adjusting the cavity thickness for the target F‐P resonant wavelength. The designed selective emitter, composed of SiO_2_ and Si_3_N_4_, effectively dissipates heat by leveraging the emissivity peak in the ATW, as confirmed by measurements.^[^ ^46^ ^]^ Consequently, this combined CRC structure successfully reduced the cooler's temperature by about 3.9 °C below ambient temperature.

With the emerging application of nanophotonic structure design by machine learning and Memetic Algorithms, Guan et al.^[^ ^101^ ^]^ developed a transmissive radiative colored cooler (transmissive CRC). It comprises a selective emitter on top and a color filter matrix at the bottom (Figure 8c). The color filter only allows light at the wavelength of the target color to be transmitted by adjusting the thickness and width of the Ag and SiO_x_ layer in it (Figure 8d). As for the selective emitter, it has high transmittance in the solar spectrum and selective thermal emission in the ATW. The selective emitter fabricated on the color filter matrix exhibits high transmittance in the solar spectrum and selective thermal emission.^[^ ^101^ ^]^ Thus, the desired transmitted color and near‐ideal selective emissivity can be achieved by stacking these two parts. Different to the planar F‐P cavity shown in Figure 8b, the lateral filling factor provides an extra degree of freedom to control the produced colors. For example, at a *h_SiOx_* thickness of 160 nm, the saturation of produced red colors gradually reduces as *w_SiOx_* width decreases from 495 to 450 nm. As *w_SiOx_* further decreases to 405 and 345 nm, yellowish and greenish hues emerge, respectively.^[^ ^101^ ^]^ According to constructive interference and effective refractive index, adjusting either the thickness or filling factor of nanocavity leads to the difference in effective optical path, thereby leading to distinct transmissive colors.

Mie resonance refers to a phenomenon that occurs when the wavelength of light is comparable to the geometric size of illuminated objects, as shown in Figure 8e. When light interacts with these objects, it causes oscillations in the electromagnetic field, leading to enhanced light scattering and absorption.^[^ ^49^, ^114^ ^]^ Mie resonance is not only useful for enhancing the solar scattering of coolers^[^ ^115^, ^116^ ^]^ but also can create structural color and achieve ink‐free printing technology^[^ ^49^, ^50^ ^]^ by selectively reflecting color. The scattering efficiency is for optimizing particle size selection. According to the Mie theory,^[^ ^117^ ^]^ the scattering efficiency *Q*_sca_ and extinction efficiency *Q*_ext_ for spheric particles can be solved by Maxwell equations:

(29) $$Q_{\mathrm{sca}}=\frac{2}{x^{2}}\sum_{n\;=\;1}^{\infty }\left(2n+1\right)\left({\left|a_{n}\right|}^{2}+{\left|b_{n}\right|}^{2}\right)$$

(30) Qext=2x2∑n=1∞2n+1Rean+bn

where *x* is the size parameter defined by the particle size, wavelength, and refractive index of the coating matrix, *a*_n_ and *b*_n_ are Mie coefficients.

CRC can be applied to building‐integrated photovoltaic (BIPV)^[^ ^51^, ^118^ ^]^ where both the aesthetic and cooling functions are required since the conventional Si photovoltaic (PV) modules have strong solar absorption and solar heating will lower the energy conversion efficiency of PV modules. As shown in Figure 8f, a functional coating comprises a non‐metallic nanoparticle, specifically a Si@SiO_2_ core‐shell nanoparticle, which contributes to the structural coloration of PV modules. PMMA was used as a matrix material and provides high thermal radiation. The Si@SiO_2_ core‐shell nanoparticle, exhibiting Mie resonance, selectively reflects visible light, thus generating a distinct structural color. As shown in Figure 8g, by changing the geometric size of the core‐shell nanoparticle, i.e., the core and shell's thickness, the reflected color and its saturation can be adjusted. The extinction behavior of plain silicon nanoparticles (*t* = 0 nm) primarily arises from Mie scattering mechanisms governed by coupled electric dipole (ED) and magnetic dipole (MD) resonances, with photon absorption playing a relatively minor role. As the core radius (*r*_core_) increases, there is a systematic redshift of the scattering peak, demonstrating that plain Si nanoparticles can achieve angle‐independent chromatic stability through wavelength‐selective Mie scattering phenomena. In these systems, structural coloration mechanisms arise from preferential backward scattering, where antiphase interference between ED and MD modes generates the desired spectral characteristics.^[^ ^51^ ^]^ Notably, silicon's intrinsic high refractive index within the visible spectrum induces moderate photon absorption, a secondary effect that synergistically enhances the thermal radiation efficiency in photonic cooling applications.

In conventional RC design, an F‐P cavity can be integrated to enhance thermal emissivity by setting the cavity thickness to more than a quarter of MIR wavelengths,^[^ ^63^, ^119^ ^]^ as used in phase‐change VO_2_ integrated structures for enlarged radiative modulation.^[^ ^76^, ^120^, ^121^ ^]^ In the context of CRC, the F‐P cavity's wavelength focus shifts to the visible spectrum, necessitating a quarter‐wave cavity thickness for specific colors.^[^ ^46^, ^47^, ^122^ ^]^ Regarding Mie resonant structures, they are widely applied in conventional RC coolers with random scatterers such as dielectric nanoparticles,^[^ ^1^, ^6^ ^]^ air pores^[^ ^62^, ^115^ ^]^ and nanofibers,^[^ ^116^, ^123^ ^]^ which are almost all optically lossless in solar wavelengths. However, to implement Mie resonance for coloration in CRC, several lossy materials like Si can be utilized.^[^ ^50^, ^51^, ^124^ ^]^ By tailoring the scatterer's structure, such as the core‐shell configuration, a relatively narrowband reflection valley can be achieved for the desired color with minimal solar absorption.^[^ ^51^ ^]^

## 4 Optical Material‐Based Coloration in CRC

Photonic structure‐based coloration can tailor structural color and is compatible with the optimization of radiative cooling performance. However, for the extensive application of CRC materials, a variety of optical materials can be facilely integrated into the CRC system to achieve coloration. High infrared emissivity can also be obtained by utilizing matrix materials with inherent molecular vibrations within the LWIR wavelength range.^[^ ^125^, ^126^ ^]^ Commercial solar‐absorptive pigments, inks, and dyes usually produce color through the selective absorption of specific wavelengths of visible light. This coloration mechanism can also be called complementary color absorption.^[^ ^36^ ^]^ The resultant color appearance is contingent upon the absorbed light spectrum. Another prominent optical material utilized for coloration is fluorescent pigments. Fluorescence is actively emitted by the material itself and is commonly observed in phosphors, light‐emitting diodes (LEDs), and bioluminescent phenomena. The coloration produced by fluorescent pigments arises from the amalgamation of visible light absorption and subsequent fluorescence re‐emission, as the fluorescence wavelength generally falls within the visible light spectrum, thereby contributing to the coloration. The principal objective of colored radiative cooling is to achieve the desired color while upholding effective cooling performance. Given the straightforward utilization of optical materials for coloration and the imperative need for scalability, it is crucial to judiciously exploit optical‐material coloration for the extensive implementation of CRC.

### 4.1 Solar‐Absorptive Pigments

Solar‐absorptive pigments can be incorporated into CRC systems to achieve different colors, which is also the technique referred to as pigmentary coloration.^[^ ^127^ ^]^ Several studies have successfully utilized particle‐embedded matrix structure configurations to create paintable CRC coatings, using practical fabrication techniques such as brushing, spraying, and blading, thereby facilitating large‐scale application.^[^ ^36^, ^37^ ^]^

**Figure 9:**
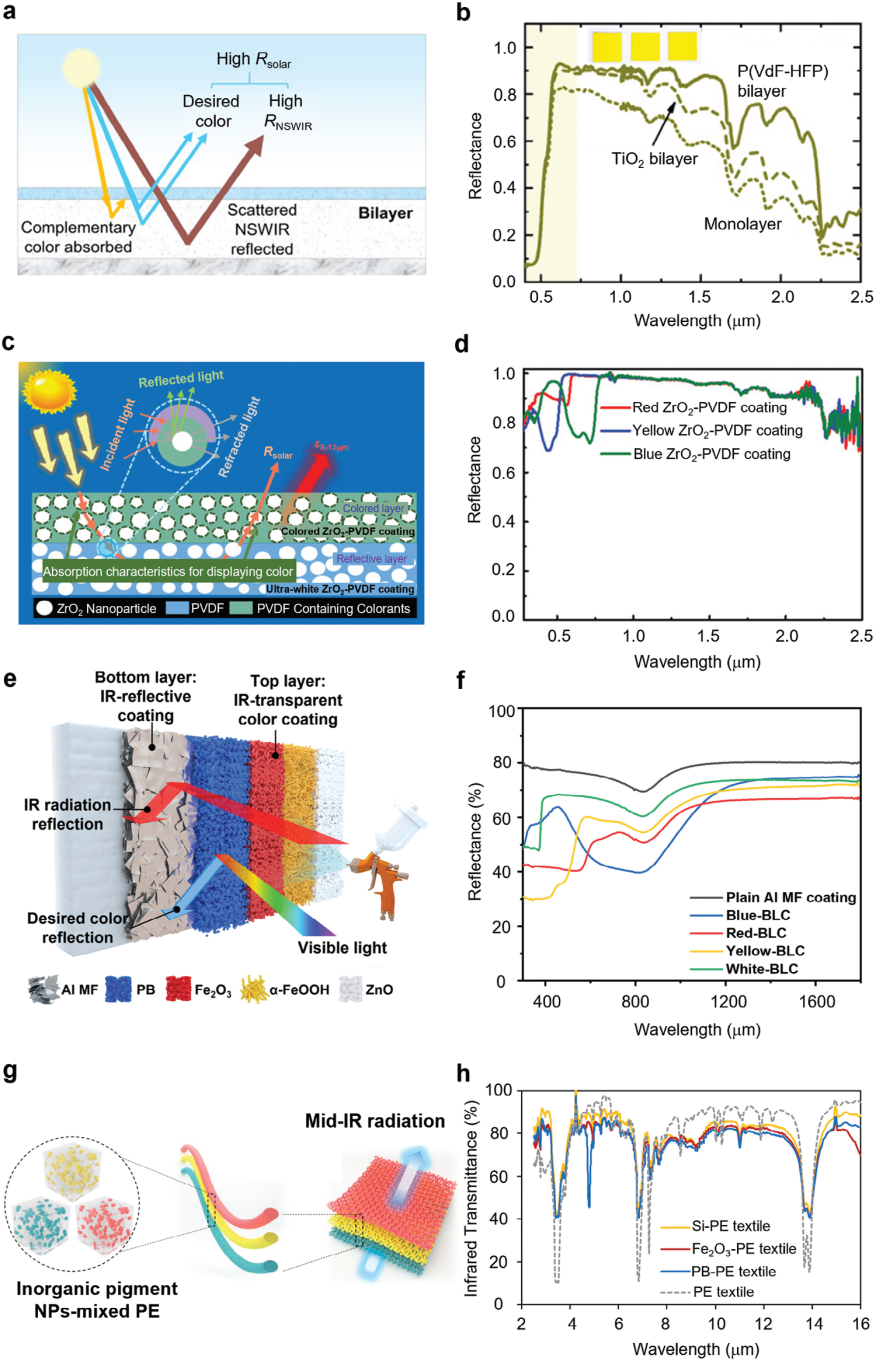
Colored Radiative Coolers with Solar‐absorptive Pigments. a) Colored bilayer coating configuration of the porous P(VdF‐HFP) CRC, TiO_2_/polymer CRC. b) Solar reflectance spectra of yellow coatings. a,b) Reproduced with permission.^[^ ^36^ ^]^ Copyright 2020, AAAS. c) Structure of bilayer‐colored radiative cooling coating with the ultra‐white bottom layer. d) Solar reflection of colored bilayer coatings. c,d) Reproduced with permission.^[^ ^128^ ^]^ Copyright 2022, Wiley‐VCH. e) Bilayer‐colored low‐E building coating configuration. f) Solar reflectivity of plain Al MF coating, Blue, red, yellow, and white BLC. e,f) Reproduced with permission.^[^ ^37^ ^]^ Copyright 2023, NAS. g) Colored inorganic pigment NPs‐mixed PE textiles. h) Infrared transmittance of PE textile with different colors. g,h) Reproduced with permission.^[^ ^129^ ^]^ Copyright 2019, Elsevier.

Chen et al.^[^ ^36^ ^]^ developed a bilayer coating that is colored and paintable using polymer matrices, TiO_2_ nanoparticles, and colorants. The top layer, as illustrated in **Figure** **9**a, consists of commercially available colorants and the polymer matrix that selectively absorbs specific wavelengths of sunlight for coloration. The underlayer comprises a porous polyvinylidene fluoride‐co‐hexafluoropropylene (PVdF‐HFP) or polymer/TiO_2_ composite coating. The function of the underlayer is to maximize the back reflectance of solar light passing through the top layer, resulting in a strong reflection of visible and NIR light (Figure 9b). Additionally, the polymer materials exhibit high LWIR emissivity, rendering the coatings effective emitters. Compared to single‐layer cooling coatings, these bilayer coatings demonstrate enhanced NIR reflectivity and can maintain lower temperatures.^[^ ^36^ ^]^ However, it is important to note the NIR absorptance of normal commercial colorants adversely affects the radiative cooling performance.

Considering minimizing solar absorption in the bilayer structure, an ultra‐white bottom layer can be chosen to reflect solar light that transmits through the topcoat effectively. As depicted in Figure 9c, Liu et al. designed a bilayer‐colored cooling coating consisting of an ultra‐white polyvinylidene fluoride (PVDF)/zirconium dioxide (ZrO_2_) bottom and a thin‐colored top layer.^[^ ^128^ ^]^ The ZrO_2_ nanoparticles size, volume fractions, and coating thickness for the PVDF/ZrO_2_ bottom layer were optimized as 200 nm, 60%, and 450 µm, respectively. This bottom exhibits an ultrahigh solar reflectance of 0.984 and LWIR emittance of 0.967. For the topcoat, only a small amount of colorant (0.05 wt%) is used to ensure a solar reflectance of > 0.9, as a high content of colorant leads to increased solar absorbance. When combined with an ultrathin‐colored top layer, the resulting colored bilayer coatings all exhibit high solar reflectance, and the solar reflectance of the optimized red, yellow, and blue colored bilayer coatings is 0.951, 0.930, and 0.905, respectively (Figure 9d), and their emittance maintains above 0.96.^[^ ^128^ ^]^

High thermal emittance is indeed a crucial performance parameter for maximizing the cooling efficiency of CRC systems. However, in specific applications such as building energy‐saving, in some climatic regions, a low‐emissivity envelope may be desirable to achieve year‐round energy savings, avoiding excessive cooling and compromising heating energy in winter.^[^ ^37^ ^]^ To address this need, Peng et al.^[^ ^37^ ^]^ introduced a colored bilayer low‐emissivity building coating, consisting of a colored and infrared‐transparent layer on top of a solar and MIR reflective bottom layer (Figure 9e). The top layer incorporates infrared‐transparent inorganic colorants such as Prussian blue (PB), Ferric oxide (Fe_2_O_3_), ferric oxyhydroxide (*α*‐FeOOH), and zinc oxide (ZnO), achieving the desired color by wideband solar absorption (Figure 9f). The bottom layer, composed of Al‐MF (Micro Flake) and polymer matrix, reflects infrared radiation and solar light that pass through the top layer. These colored bilayer coatings (BLC) attain a high solar reflectance while maintaining the desired color and exhibit low MIR emissivity.

Advanced colored textiles have also been investigated for personal thermal management, which requires high infrared transparency or high emission properties to transmit thermal radiation from the human body. Figure 9g illustrates the structure of a colored inorganic pigment/polyethylene (PE) composite textile.^[^ ^129^ ^]^ Both the selected inorganic pigments and the PE matrix exhibit infrared transparency, allowing for high MIR radiation transmission from the human body through the textile. This is corroborated by the high infrared transmittance observed in the textile (Figure 9h). Additionally, PE textiles mixed with inorganic pigment nanoparticles demonstrate excellent mechanical properties under tension and abrasion loads.^[^ ^129^ ^]^ Notably, even after 120 wash cycles, the mass loss of the pigments remains below 4%.

To summarize, commercial colorants generally exhibit broadband solar absorption characteristics. Therefore, to achieve optimal cooling performance, it is imperative to use a very thin colored top layer and colorants with low‐volume fractions. This strategy allows for effective coloration while mitigating the adverse impact of solar‐absorptive pigments on radiative cooling performance. An alternative approach to reducing solar absorption by pigments is to hybridize them with high concentrations of light‐scattering dielectric nanoparticles, enabling relatively high solar reflectance with a monolayer coating. However, the substantial volume fraction of these nanoparticles may lead to low color saturation, potentially failing to satisfy aesthetic preferences in certain applications.

### 4.2 Fluorescent Pigments

Solar‐absorptive pigments generate color through selective absorption of visible light, resulting in a considerable increase in heat load. To mitigate this issue, introducing fluorescence is preferable as it can convert absorbed excitation light and re‐emit fluorescence, thereby reducing solar heat accumulation. Common types of down‐conversion photoluminescent materials include inorganic rare‐earth‐doped phosphors^[^ ^130^ ^]^ and fluorescent pigments with long decay lifetimes,^[^ ^23^ ^]^ organic fluorescent dye,^[^ ^131^ ^]^ and quantum‐dot materials.^[^ ^56^, ^81^ ^]^ Down‐convention fluorescent pigments are typically excited by UV or short‐wavelength light, such as blue and green light, and re‐emit photons at longer wavelengths, usually within the visible range. The photon conversion efficiency can be defined as the proportion of the overall emitted photon energy at the emission wavelength to the total photon energy absorbed. The theoretical calculation of the photo conversion efficiency (ε(*λ*)) relies on the absorption and emission wavelengths and can be determined using the subsequent equation:^[^ ^81^ ^]^

(31) $$\varepsilon \left(\lambda \right)=\left(\frac{\lambda_{\mathrm{abs}}}{\lambda_{\mathrm{em}}}\right)\Phi \left(\lambda \right)$$

where *λ*_abs_ represents the wavelength at which absorption occurs, Φ is the photoluminescence quantum yield (PLQY): 0 ≤ Φ ≤ 1, and λ_em_ indicates the wavelength of the emitted light. Larger PLQY leads to higher photon conversion efficiency. Moreover, a small Stoke shift ratio ($\frac{\lambda_{\mathrm{abs}}}{\lambda_{\mathrm{em}}}$) can avoid large energy loss during photon conversion. Therefore, both higher PLQY and smaller Stoke Shift can reduce the heat load during photoluminescence conversion and thus improve the radiative cooling performance, which has also been validated by experiment study.^[^ ^39^ ^]^

**Figure 10:**
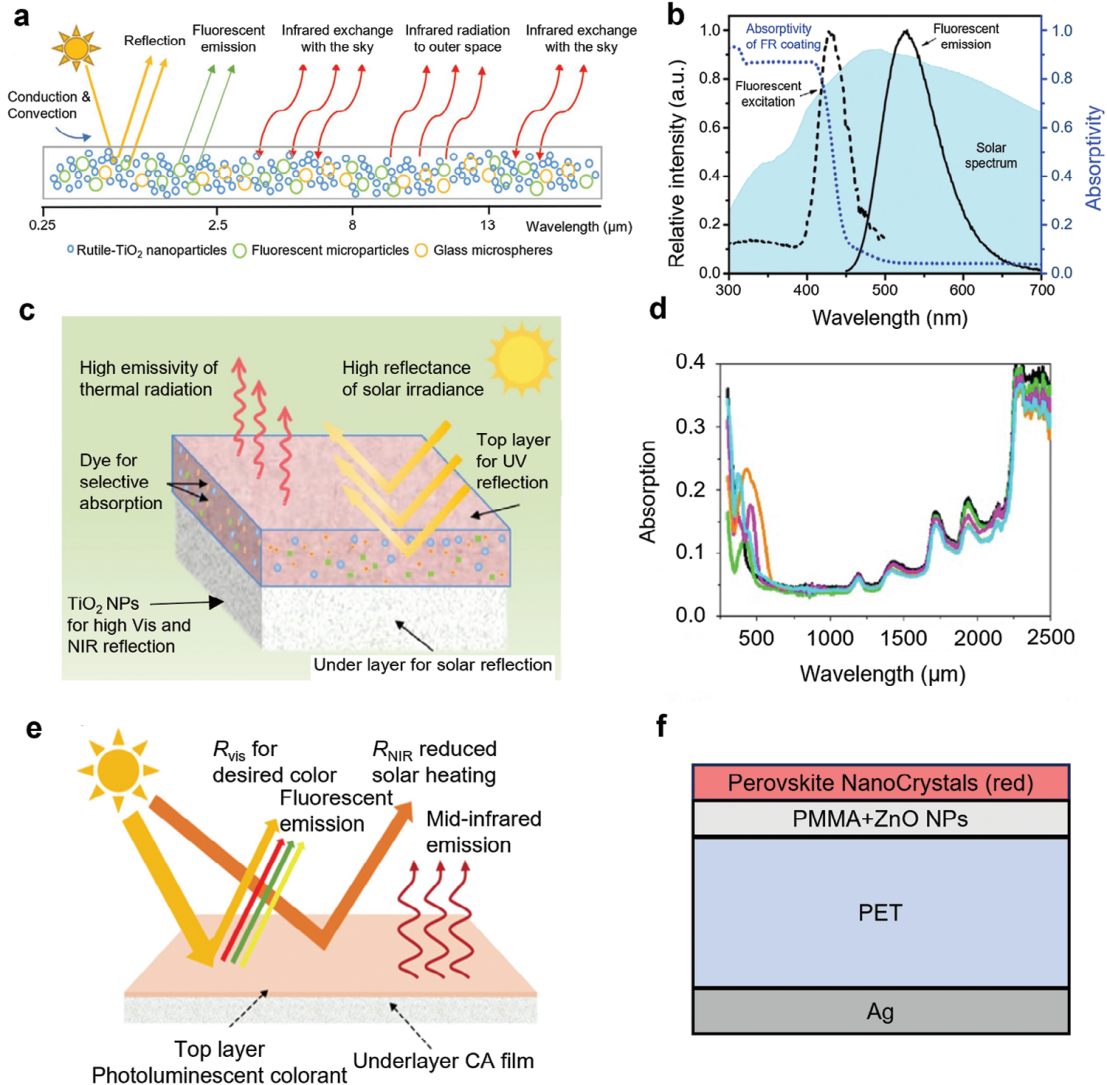
Colored Radiative Coolers with Fluorescence. a) Structure of fluorescence‐colored ecofriendly radiative cooling coating. b) Excitation and emission spectrum of used fluorescent pigment. a,b) Reproduced with permission.^[^ ^23^ ^]^ Copyright 2020, Wiley‐VCH. c) Structure configuration of colored cooling coatings with phosphor. d) Absorption spectra of phosphor‐colored cooling coatings. c,d) Reproduced with permission.^[^ ^130^ ^]^ Copyright 2022, Elsevier. e) Structure configuration of colored bilayer cooling coatings with perovskite quantum dot. Reproduced with permission.^[^ ^81^ ^]^ Copyright 2022, Elsevier. f) Structure of the colored cooler with perovskite nanocrystals. Reproduced with permission.^[^ ^56^ ^]^ Copyright 2021, Elsevier.

Regarding the color produced by fluorescent radiative coolers, it is important to note that re‐emitted fluorescence also contributes to color formation, in addition to partial visible light absorption when using fluorescent pigments. Several research works have explored the utilization of fluorescence‐enhanced colored radiative cooling, capitalizing on the energy conversion properties inherent in fluorescent pigment materials. TiO_2_ nanoparticles are frequently utilized in solar‐reflective coatings due to their high refractive index^[^ ^132^ ^]^ (over 2.4) and effective backscattering ability. About 4% volume fraction of TiO_2_ in PDMS can lead to a solar reflectance of ≈0.89 while maintaining reasonable workability for coating fabrication. However, TiO_2_ absorbs UV due to its low bandgap of ≈3 eV. To address this issue, photoluminescence was introduced to compete with the UV absorption from TiO_2_. Xue et al.^[^ ^23^ ^]^ developed a sub‐ambient daytime radiative cooling coating by combining TiO_2_ nanoparticles, hollow glass microsphere with UV‐excited fluorescent pigments(SrAl_2_O_4_:Eu^2+^,Dy^3+^,Yb^3+^), as depicted in **Figure** **10**a. In the unexcited state, coating with and without fluorescent pigments exhibited averaged solar reflectance of 0.898 and 0.895, respectively.^[^ ^23^ ^]^ Figure 10b illustrates the excitation peak of the fluorescent pigment is about 420 nm, coincidently below the absorption wavelength range of TiO_2_. However, when illuminated by solar light, the fluorescent pigments within the coatings can effectively convert part of the absorbed solar light (below 450 nm) into visible fluorescence and re‐emission. Furthermore, the fluorescent pigment surrounded by TiO_2_ nanoparticles forms a Purcell cavity, which accelerates the emission rate of the converted photons. This phenomenon resulted in an improved effective solar reflectance and produced yellow‐green luminescence. Xu et al.^[^ ^130^ ^]^ reported a bilayer‐colored cooling coating consisting of a phosphor‐colored top layer and a solar‐reflective bottom layer (Figure 10c). The top layer contains BaSO_4_ nanoparticles and phosphor dyes, mainly for UV and visible reflection, as well as narrowband absorption (Figure 10d) for the target color with the fluorescence emission. This coating also exhibits good LWIR infrared emittance of ≈0.93 and achieves a sub‐ambient effect of ≈0.7 °C temperature reduction for the red coating.^[^ ^130^ ^]^

Furthermore, additional fluorescent materials, such as perovskite quantum dots, can also be incorporated into the radiative cooling coating system. Wang et al.^[^ ^81^ ^]^ designed a bilayer‐colored radiative cooling cooler with a perovskite quantum dot layer atop highly solar‐reflective cellulose acetate film (Figure 10e). Since perovskite quantum dots can be excited and emit photons via down‐conversion, they positioned it as the top layer to achieve the desired color while minimizing the heat load from selective visible light absorption. Son et al.^[^ ^56^ ^]^ developed a colored radiative cooler with a multilayer structure, consisting of perovskite nanocrystals at the top, followed by a hybrid layer of PMMA/ZnO nanoparticles, polyethylene terephthalate (PET), and Ag layers, depicted in Figure 10f. The solar reflector consists of Ag and PMMA/ZnO nanoparticle layers, while the top layer comprises silica‐embedded perovskite nanocrystals, serving as the chromogenic layer. The chromogenic layer contains a relatively high concentration of perovskite nanocrystals, allowing this composite material to achieve a specific color appearance. Additionally, the photoluminescent behavior of perovskite nanocrystals efficiently reduces thermal load and significantly improves the cooling performance during daylight hours. The fabricated CRC materials, available in various colors, demonstrated sub‐ambient temperature reduction ranging from 1.7 to 4.2 °C.^[^ ^56^ ^]^

When using fluorescent material in CRC design, an important parameter to consider is the photoluminescent quantum yield (PLQY), representing the ratio of reemitted photons to excited photons.^[^ ^81^ ^]^ A higher PLQY leads to more converted photons and less non‐radiative heat generation.^[^ ^39^ ^]^ PLQY becomes increasingly significant in compensating for solar heat when higher concentrations of fluorescent pigments are used.^[^ ^80^ ^]^ In addition, the photon conversion process can be enhanced through Purcell enhancement,^[^ ^23^, ^39^ ^]^ when abundant dielectric nanoparticles are located in the vicinity of fluorescence pigments, the resulting shell formed by these dielectric nanoparticles is in analogy to a Purcell cavity which can enhance and accelerate the photon emission rate of the fluorescent material. By modifying the surrounding dielectric environment to match the emission peak of the fluorescent pigment and the scattering peak of the surrounding nanoparticles, the PLQY can be increased by photonic structure‐induced electromagnetic field enhancements such as the Purcell cavity.^[^ ^39^, ^80^, ^133^ ^]^ Moreover, since the inherent UV conversion process of down‐conversion type fluorescent material, it holds the potential to weaken the UV aging process of colored coatings through UV absorption in competition with the polymer matrix. Furthermore, great emphasis should be placed on discovering and developing fluorescent materials with narrow excitation wavelength ranges and high PLQY to achieve vivid coloration and enhanced cooling capabilities in CRC materials. Improvements are needed in the fluorescent properties, environmental stability, and fabrication processes of these materials. This includes the careful selection and doping of rare‐earth elements for inorganic phosphors,^[^ ^134^, ^135^ ^]^ as well as advancements in synthesis processes and photoluminescence enhancement strategies for durable perovskites.^[^ ^136^, ^137^ ^]^

## 5 CRC with Dynamic Modulation

**Figure 11:**
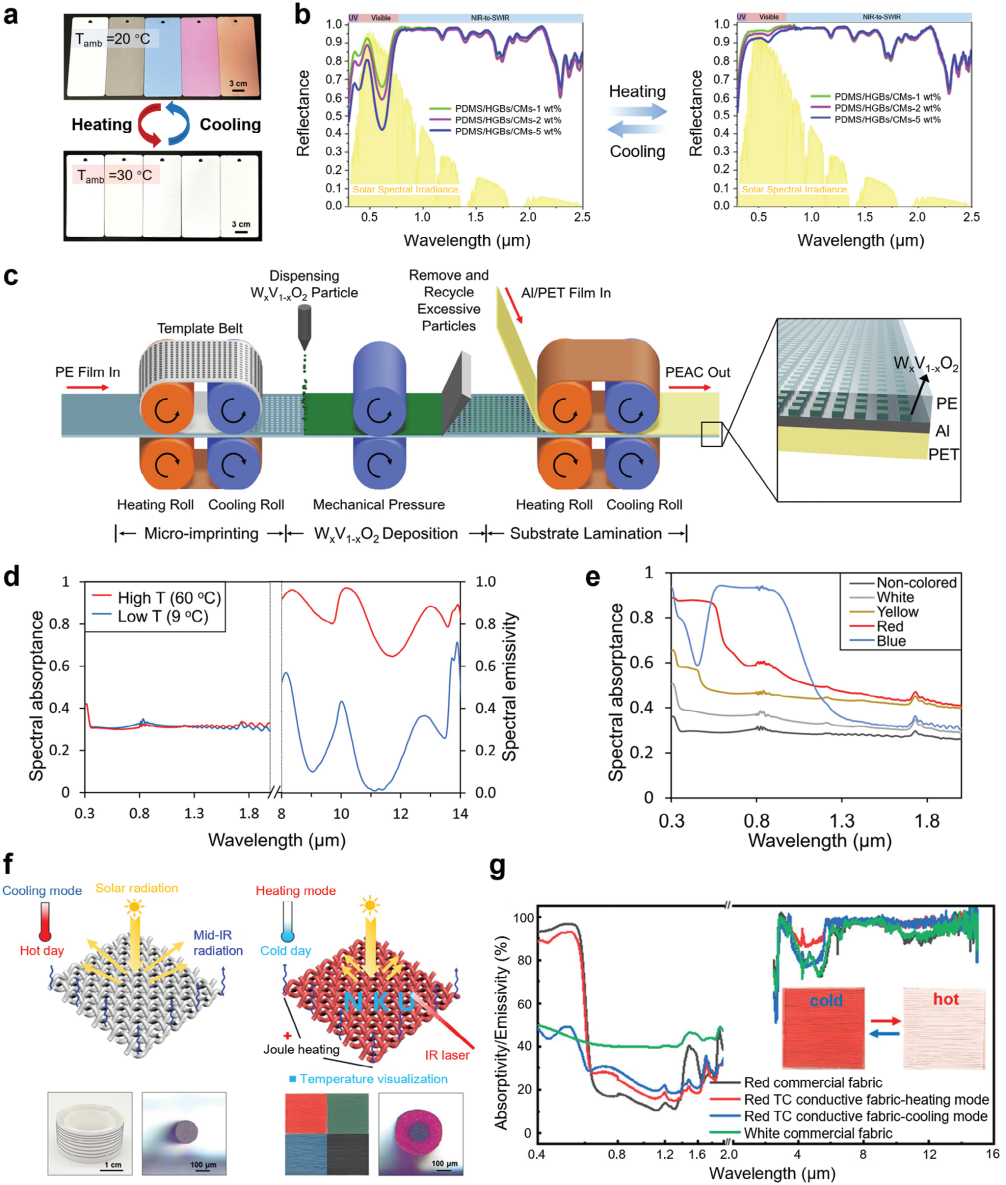
Colored Radiative Cooling with Dynamic Modulation. a) Color change of coatings between heating and cooling modes. b) Reflectance spectra of coatings between heating and cooling modes. a,b) Reproduced with permission.^[^ ^25^ ^]^ Copyright 2022, Elsevier. c) Fabrication and structure of printable, emissivity‐adaptive, and albedo‐optimized covering. d) Solar absorptance and infrared emissivity spectra of the non‐colored covering. e) Absorptance spectra of colored fabricated coverings. c–e) Reproduced with permission.^[^ ^61^ ^]^ Copyright 2023, Elsevier. f) Schematic of cooling mode and heating mode of colored thermochromic conductive textile. g) Absorptivity and infrared emissivity spectra of red thermochromic textiles compared with commercial fabric. f,g) Reproduced with permission.^[^ ^27^ ^]^ Copyright 2023, ACS Publication.

Compared to their white counterparts, CRC materials inevitably compromise cooling performance due to reflection loss in the visible spectrum necessary for color appearance. In addition, considering seasonal temperature variations and diurnal temperature differences, overcooling has emerged as an issue for radiative cooling applications, offsetting energy savings and harming the service life of buildings and other infrastructure.^[^ ^23^ ^]^ Therefore, introducing the self‐adaptive properties to CRC materials is essential to achieve intelligent energy‐saving and thermal management. Thermochromic pigments have been used to create colored radiative cooling materials with dynamic modulation properties.^[^ ^25^, ^26^, ^138^, ^139^, ^140^ ^]^ Reversible thermochromic materials can be categorized into three main groups: organic materials, inorganic materials, and liquid crystals.^[^ ^59^ ^]^ Charge‐transfer‐based organic thermochromic microcapsules have gained significant interest due to their various and easily adjustable discoloration temperature, extensive color choices, prominent discoloration effect, high transition sensitivity, and cost‐effectiveness.^[^ ^59^, ^141^, ^142^, ^143^ ^]^ The thermochromic microcapsule consists of a wall material and a core part that includes a thermochromic organic dye, a color developer, and a solvent.^[^ ^59^ ^]^ At lower temperatures, the thermochromic organic dye accepts electrons from the color developer, displaying a specific color. As temperature rises, the solvent mixture gradually melts, causing the color developer to dissolve and separate from the dye, resulting in a molecular configuration change of the thermochromic organic dye. Consequently, the color of the microcapsule fades. Therefore, thermochromic microcapsule‐based pigments can exhibit a critical temperature (*T*_c_) at which a phase transition occurs. Beyond the *T*_c_, the pigment transitions from a colored state to a white or colored state, thereby decreasing solar absorption. Conversely, below *T*_c_, the pigment reverts to its colored state and thus gains more solar heat. A PDMS‐based thermochromic colored radiative cooling coating was developed by mixing with hollow glass microspheres and thermochromic microcapsules in the acrylic resin matrix.^[^ ^25^ ^]^ This coating can be fabricated via scalable brushing techniques. The hollow glass microsphere enhances the scattering of solar light, while the thermochromic microcapsules endow the coating with cooling and solar heating switching capabilities. As shown in **Figure** **11**a, the color change of this coating happens when its temperature jumps between *T*_c_. Figure 11b shows a solar reflectance switching happens between heating and cooling modes, resulting in a temperature difference of 9.5 °C between these two modes.^[^ ^25^ ^]^

Except for using thermochromic microcapsule‐based pigments for dynamic solar reflection modulation, other phase‐changing materials like vanadium dioxide (VO_2_) and tungsten(W)‐doped VO_2_ can be utilized for thermal radiation modulation. VO_2_ is a compound that undergoes a fascinating phase transition known as the metal‐insulator transition at a transition temperature of ≈68 °C. By adjusting the amount of doped W in VO_2_, the phase‐changing temperature can be tailored to around ambient temperature such as 30–35 °C, to meet real application needs.^[^ ^76^ ^]^ In its low‐temperature state, VO_2_ behaves as an insulator with a monoclinic crystal structure. In its high‐temperature state, it becomes a metal with conductive properties, undergoing a remarkable transformation from the dielectric monoclinic phase to the metallic rutile phase.^[^ ^144^ ^]^ Leveraging VO_2_ thermochromic material, Li et al. designed a colored W_x_V_1‐x_O_2_‐based printable, emissivity‐adaptive, and albedo‐optimized covering (PEAC).^[^ ^61^ ^]^ As shown in Figure 11c, the covering can be fabricated via the roll‐to‐roll technique for large‐scale applications. Figure 11c (right inset) illustrates the structure configuration of the noncolored covering before incorporating IR‐transparent pigments into pristine thermochromic covering. It comprised four parts from top to bottom: an IR‐transparent PE layer, a W_x_V_1‐x_O_2_ block array interlocked above the PE layer, a bottom Al layer, and a PET layer. Figure 11d shows that the covering exhibits a significant emissivity difference between heating and cooling modes, while the solar absorptance remains almost unchanged. IR‐transparent pigments are incorporated to generate color appearance via selective visible absorption while slightly increasing the heat load (Figure 11e). This has a marginal effect on the emissive‐adaptive performance of PEAC since both PE and pigments are MIR transparent material. Adjacent W_x_V_1‐x_O_2_ blocks act as optical antennas, effectively amplifying the electric field intensity on their surfaces and generating strong evanescent waves absorbed within the metallic W_x_V_1‐x_O_2_ blocks.^[^ ^61^ ^]^ As a result, the periodic arrangement of W_x_V_1‐x_O_2_ blocks forms an array of lossy‐effective optical antennas, leading to high MIR absorptance. The underlying Al layer reflects transmitted solar and MIR light, contributing to the low emissivity of PEAC under cold weather conditions. PEAC is capable of automatically switching the LWIR emissivity from ≈0.25 to ≈0.85 when the temperature of PEAC reaches the transition temperature.^[^ ^61^ ^]^

In addition to the above colored thermochromic building envelopes for energy‐saving, colored thermochromic textiles have also drawn people's attention to their potential application for personal thermal management. Yu et al.^[^ ^27^ ^]^ reported a colored thermochromic conductive textile for advanced personal thermal management with an additive temperature visualization function (Figure 11f). The colored conductive fiber utilized in the textile comprises an Ag nanoparticle‐based polyurethane core and a thermochromic microparticle‐based waterborne polyurethane shell (Figure 11f). Its color can change from color to white, creating a solar reflectance difference between heating and cooling modes (Figure 11g), akin to the thermochromic coatings discussed earlier. Leveraging the excellent electrical conductivity of the core, this TC conductive fabric can generate Joule heat in cold conditions.^[^ ^27^ ^]^ Integrating Joule heating and photothermal effects in typical thermal management technologies maximizes the unique benefits of each method, leading to enhanced temperature control and improved overall efficiency.

Conventional thermochromic pigments, such as charge‐transfer‐based organic microcapsules, mainly adjust visible light absorption and exhibit color‐changing properties. By integrating phase‐change materials like pure VO_2_ or W‐doping VO_2_ into CRC designs, it is possible to modulate NIR reflection and thermal emittance without altering color. Nonetheless, exploring alternative spectral modulation methods or combining multiple modulation mechanisms, such as electrochromic,^[^ ^145^, ^146^ ^]^ photochromic,^[^ ^147^, ^148^ ^]^ and mechanochromic technologies,^[^ ^149^, ^150^ ^]^ is essential for broadening the range of dynamic CRC materials. Designing corresponding photonic structures can achieve multispectral modulation for advanced smart CRC materials.

## 6 Application of CRC Materials

**Figure 12:**
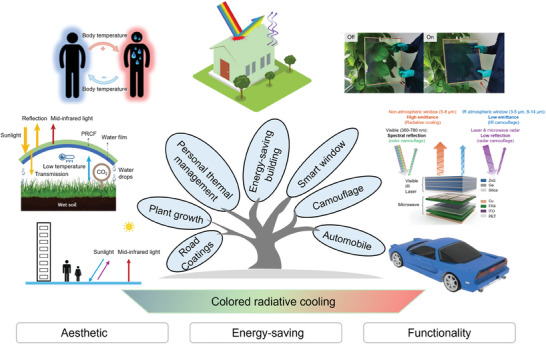
Summary of the colored radiative cooling in diverse applications, including road coatings. (reproduced with permission.^[^ ^176^ ^]^ Copyright 2024. Springer Nature), plant growth (reproduced with permission.^[^ ^30^ ^]^ Copyright 2024. Springer Nature), personal thermal management (reproduced with permission.^[^ ^70^ ^]^ Copyright 2021. Springer Nature), building roof/walls (reproduced with permission.^[^ ^71^ ^]^ Copyright 2024. ACS Publication), smart window (reproduced with permission.^[^ ^145^ ^]^ Copyright 2022. Springer Nature), camouflage (reproduced with permission.^[^ ^29^ ^]^ Copyright 2021. Springer Nature.) and automobile (reproduced with permission.^[^ ^68^ ^]^ Copyright 2022. Springer Nature). Colored radiative cooling materials can enhance these applications with aesthetics, energy‐saving, and functionalities.

When the colored aesthetic requirement is fulfilled without significantly compromising cooling performance, CRC materials hold substantial potential for diverse applications, including building envelopes, textiles, camouflage, plant growth, roads, and vehicles, as shown in **Figure** **12**. These materials demonstrate significant value in energy conversion and sustainability.

### 6.1 Building Roof/Walls

Nearly 40% of total energy consumption occurs in buildings.^[^ ^151^, ^152^ ^]^ Integrating radiative cooling technology into buildings significantly enhances energy savings and reduces greenhouse gas emissions. The plain white or silver color does not meet aesthetic standards or provide visual comfort, and it may even contribute to light pollution. Hence, developing colored radiative cooling materials for energy‐efficient buildings is crucial for broadening applications and meeting functional requirements. Given that the visible spectral region (380−780 nm) accounts for a substantial part of the solar radiation region, fine‐tuning reflectance within this range is vital for achieving color options while effectively preserving cooling performance.

Optimizing radiative cooling performance for colored building roofs requires maximizing reflectance in the UV and NIR spectral regions to block solar heating while achieving exceptionally high emissivity in the LWIR spectrum to effectively radiate heat to the cold sky.^[^ ^36^, ^80^ ^]^ Conversely, for colored building walls, where radiative heat exchange with outdoor ambient surroundings is more pronounced than with the sky, maintaining high reflectivity in the thermal infrared range proves advantageous for energy savings year‐round. During summer, this reduces thermal infrared radiative heat gain from hot surroundings, while in winter, it minimizes thermal infrared radiative heat loss to cold surroundings, thus effectively conserving energy for both cooling and heating throughout the year while preserving the desired color.^[^ ^37^, ^153^ ^]^ Moreover, angle‐selective radiators that exhibit high emissivity towards the sky but low emissivity towards the hot ground,^[^ ^154^ ^]^ and LWIR selective emitters,^[^ ^155^ ^]^ have been demonstrated to produce a superior cooling effect on walls compared to conventional broadband emitters. This is attributed to the reduced radiation heat from the ground.

Beyond meeting aesthetic demands, buildings necessitate the maintenance of thermally comfortable indoor environments across diverse climatic scenarios. Incorporating switchable radiative cooling technology into buildings can achieve both winter warmth and summer cooling, thereby ensuring thermal comfort year‐round while also promoting energy efficiency, The radiative coolers can transition from a colored state at low temperatures to a white state at higher ambient temperatures by integrating reversible thermochromic chameleon microcapsules. This allows the coatings to dynamically provide cooling by radiating heat away in hot conditions and absorbing solar heat, warming the space in cooler conditions.^[^ ^25^, ^60^ ^]^

An additional promising avenue involves fine‐tuning their thermal emittance in response to fluctuating ambient temperatures. Wu et al. developed temperature‐adaptive radiative coating (TCRC) leveraging the metal–insulator transition (MIT) of W_x_V_1‐x_O_2_ materials, offering a near 0.7 of radiative cooling modulation. By embedding a lithographically patterned array of W_x_V_1‐x_O_2_ blocks in a barium fluride (BaF_2_) dielectric layer atop a silver film, the TCRC coating could automatically switch thermal emittance from 0.20 for ambient temperatures lower than 15 °C to 0.90 for temperatures above 30 °C.^[^ ^76^ ^]^ Further advancement in scalability was achieved through roll‐to‐roll fabrication using W_x_V_1‐x_O_2_ and inexpensive recyclable materials.^[^ ^61^ ^]^ To achieve maximum energy efficiency across varying climatic conditions, the TCRC can be customized utilizing mid‐infrared transparent pigments to optimize visual aesthetics across a spectrum of colors. Furthermore, the substitution of the thermal infrared‐reflective silver or aluminum layer with transparent conducting oxides enables the fabrication of optically transparent TCRC, opening opportunities for use in window coatings.

### 6.2 Colored Smart Windows

Windows serve as the primary interface for energy exchange between interior and exterior environments, significantly impacting building energy consumption by accounting for up to 20% of HVAC (heating, ventilation, and air conditioning) energy loss.^[^ ^145^ ^]^ The integration of color is pivotal in enhancing solar modulation capacity in high‐performance smart windows, such as those incorporating perovskite‐based thermochromic elements,^[^ ^156^, ^157^ ^]^ and electrochromic components capable of colored‐to‐bleached switching.^[^ ^158^, ^159^ ^]^ In the cold state, the window maintains a highly bleached/transparent appearance, while it transitions to a colored state to markedly reduce solar transmittance through broad‐spectrum solar absorption in the hot state. However, LWIR thermal radiation should also be considered in the multispectral design of advanced colored smart windows. The inclusion of photonic structures, such as F‐P cavities in VO_2_‐based thermochromic smart windows with a light‐yellow hue, can enhance LWIR emissivity modulation.^[^ ^113^, ^160^ ^]^ These structures exhibit high thermal emission during hot seasons for cooling while switching to low thermal emissivity in cold seasons to minimize heat loss. Challenges persist in developing colored‐to‐bleached‐switching smart windows with radiative modulation to further optimize energy conservation efficiency. Shao et al. designed a tri‐band electrochromic smart window with a tungsten oxide (WO_3_)/VO_2_ film structure, displaying high solar transmittance in the cold state and low solar transmittance with varied coloring in the hot state by adjusting input voltages.^[^ ^161^ ^]^ This electrochromic smart window also exhibits high thermal emittance on the exterior surface and low emissivity on the interior surface, demonstrating superior energy conservation compared to conventional glass windows in building‐scale simulations.

### 6.3 Personal Thermal Management

Unlike building‐level temperature regulation, which often results in significant energy waste in unoccupied spaces, personal thermal management offers a more efficient and cost‐effective alternative by targeting localized heating and cooling to the human body and its immediate surroundings. Colored radiative cooling materials present an innovative, energy‐efficient solution for personal cooling, improving thermal comfort while meeting aesthetic preferences. Human skin, acting almost like a perfect black body, emits thermal radiation primarily in the infrared wavelength range of 7–14 µm, peaking at ≈9.5 µm according to Planck's law.^[^ ^10^ ^]^ In addition to selective reflection in the visible spectrum for achieving desired colors and high UV and near‐infrared reflectance, the materials should exhibit high emissivity^[^ ^162^ ^]^ or high transmittance^[^ ^129^ ^]^ in the mid‐infrared range. This ensures that thermal radiation from warm human skin can either be emitted or pass through the textile, directly reaching the surrounding indoor environment or the cold outer space when outdoors. IR transparent materials including polyethylene and IR‐transparent pigments are often utilized in colored transmission‐type textiles.^[^ ^129^, ^163^ ^]^ In addition, an adaptive textile was tailored to exhibit emission‐type characteristics in the atmospheric transparency window and transmission‐type characteristics outside the window, making it with an optimal emissive cooling effect for both outdoor and indoor environments.^[^ ^9^ ^]^ To address diverse environmental conditions and thermal requirements, there is a pressing need for the advancement of dynamic radiative cooling/heating textiles.^[^ ^164^, ^165^ ^]^ Moreover, the integration of radiative thermal engineering with other textile‐based heat management mechanisms, such as heat conduction and evaporative heat dissipation, offers a promising avenue for achieving multimodal control over thermal dynamics, thus advancing the effectiveness of personal thermal management.^[^ ^70^, ^166^ ^]^ From a practical perspective, in addition to spectral management requirements, colored radiative cooling materials must also possess wear resistance, breathability, comfort, scratch resistance, and washability.

### 6.4 Camouflage Technologies

Camouflage materials are especially crucial for enhancing the survivability of the military by minimizing their detectability across multiple spectral bands, thereby evading diverse detection systems such as thermal imagers, radar, and lasers.^[^ ^29^, ^167^, ^168^, ^169^, ^170^ ^]^ Different spectral bands necessitate distinct material properties for effective camouflage. For instance, materials must exhibit characteristic reflection within the visible range (380−780 nm) to ensure background matching, and high absorbance in the microwave radar band (X‐band, 8–12 GHz) to reduce radar cross‐section. Additionally, high absorbance at specific laser wavelengths (1.55 and 10.6 µm) is required. Effective camouflage should also exhibit low emissivity in the mid‐wavelength infrared range of 3–5 µm and the long‐wavelength infrared range of 8–14 µm to counter thermal imagers and heat‐seeking missiles. Additionally, IR camouflage performance can be enhanced by lowering the surface temperature through radiative heat dissipation, which requires high emissivity within the MIR non‐atmospheric window (5‐8 µm) to facilitate radiative cooling and reduce heat load.

### 6.5 Plant Growth

Plant growth is fundamentally linked to photosynthesis, the process by which plants convert light energy into chemical energy to fuel their growth and development. For optimal photosynthesis, plants require light in the photosynthetically active radiation range, which spans wavelengths from 400 to 700 nm. Within this range, blue light (400–500 nm) and red light (600–700 nm) are most effective, as they are strongly absorbed by chlorophyll, the primary pigment involved in photosynthesis. Adequate light quality and quantity are crucial for maximizing photosynthetic efficiency, thereby promoting healthy plant growth and higher crop yields.^[^ ^31^ ^]^ Moreover, reducing the ambient air temperature to minimize water evaporation can also promote the survival and growth of plants in arid regions. Colored radiative cooling materials tailored to support photosynthesis while mitigating heat should effectively transmit sunlight in the specific ranges of 0.4–0.5 µm and 0.6–0.7 µm, reflecting the remaining part of the solar spectrum. Additionally, these materials should demonstrate heightened emissivity within the mid‐infrared spectrum (2.5–20 µm), facilitating efficient heat dissipation into the surrounding environment and outer space.^[^ ^30^ ^]^ The significance of designing colored radiative cooling modules to promote plant photosynthesis lies in their ability to sequester atmospheric carbon dioxide, aiding in mitigating climate change while providing additional ecological benefits.

### 6.6 Road Coatings

The rapid development and expanding urbanization of cities are substantially affecting ecosystems and climate, with road surface temperature changes being a pivotal factor. Elevated energy consumption in urban environments directly contributes to higher road surface temperatures. Consequently, the urban heat island effect, driven by anthropogenic activities and urbanization‐induced temperature rise, has become a critical urban issue. This phenomenon results in increased energy demand, degraded air quality, and heightened health risks, underscoring the urgent need for advanced materials and innovative solutions to mitigate these impacts.^[^ ^6^, ^171^ ^]^ In recent decades, the use of cool materials, including high‐reflective and retro‐reflective coatings, has been proposed to alleviate the urban heat island.^[^ ^172^ ^]^ Additionally, light colors are preferred to be incorporated into road cooling coatings for anti‐glare requirements and driver safety.^[^ ^173^, ^174^, ^175^ ^]^ Specifically, retro‐reflection, a unique type of light reflection where the reflected light travels to the source along the incident light's path, aids in reducing glare resulting from complex light reflections. A study has demonstrated that retro‐reflective walls and pavements can reduce surface temperatures within urban canyons by as much as 20 °C and lower canyon air temperatures by up to 2.6 °C. These surfaces surpass highly reflective alternatives, resulting in significant enhancements in pedestrian thermal comfort, including reductions in human skin temperature by up to 0.55 °C and net radiative gain by 153 W m^−2^.^[^ ^176^ ^]^ In addition to offering visual appeal, thermochromic coatings applied to pavements for dynamic thermoregulation exhibit enhanced cooling effects surpassing those of traditional cool coatings.^[^ ^140^ ^]^

### 6.7 Automobile

During daylight hours, the interior temperature of automobiles can rapidly increase due to solar radiation, often exceeding 50 °C under strong sunlight. Cooling the automobile to a comfortable driving temperature using air conditioning requires substantial energy. Given the global fleet of 1.474 billion vehicles, identifying an energy‐efficient cooling solution is critical. This need is particularly pressing for the growing number of electric vehicles, where high interior temperatures can degrade battery performance and pose significant safety hazards.^[^ ^177^ ^]^ When purchasing a car, color remains a significant consideration for many buyers, often reflecting their personality and preferences. Those seeking to stand out might choose bright, bold colors like yellow, red, or green, while individuals preferring simplicity may opt for white, silver, gray, or black. Therefore, developing colored radiative cooling technology that balances optimized cooling performance with aesthetic appeal can significantly advance its commercialization in the automotive industry.^[^ ^22^, ^162^, ^178^ ^]^ It is worth noting that for a vehicle where the internal temperature exceeds that of the roof, a Janus emitter has been suggested, which functions as a selective emitter on the upper side and a broadband emitter on the lower side. This configuration efficiently extracts heat from both the inner space and the surface, as the lower side absorbs thermal energy across a wide spectral range, while the upper side radiates heat to space without interfering with ambient radiation.^[^ ^16^ ^]^ Incorporating passive radiative cooling into pre‐existing actively temperature‐regulated and aesthetically designed enclosures could provide an additional avenue for reducing active power consumption.^[^ ^68^ ^]^

## 7 Conclusions and Perspectives

This article provides a comprehensive review of CRC technologies. Initially, we systematically introduce the principles of colored radiative cooling and methods for characterizing color. Subsequently, we classify and analyze photonic structures for generating color based on utilized various photonic approaches, which aim to reduce solar absorption and enhance thermal emission. These methods encompass Fabry‐Pérot resonance, plasmon resonance, Mie resonance, photonic crystals, and iridescent periodic interference structures. Diverse photonic radiative coolers incorporating structural color have been designed and constructed, demonstrating efficient cooling performance and high infrared thermal radiation. Additionally, optical materials such as commercial solar‐absorptive pigments and fluorescent pigment materials can be directly integrated into the CRC system to achieve the desired coloration. By regulating the concentration of colorants utilized, boosting the fluorescence component to enhance effective solar reflectance, and configuring bilayer coatings, efficient colored radiative cooling can be attained. We also review advancements in thermochromic CRC featuring dynamic spectral modulation properties and the application of CRC materials in various fields. We have compiled a summary of representative CRC materials in **Table** **1** to enable a fair to facilitate a fair comparison of their color and cooling properties. Furthermore, **Table** **2** offers a comparative analysis of two‐color categories within CRC materials. The methods for implementing coloration through photonic structures and optical materials differ significantly. Achieving vibrant color in CRC coolers with high cooling capacity remains challenging. Color vibrancy is typically quantified by the color saturation index, where higher saturation indicates a denser color. For fluorescent CRC coolers, high fluorescent emission enabled by elevated PLQY enhances color saturation and improves cooling. The appropriate excitation wavelength range determines color chroma. Greater absorption within this range increases color saturation but also boosts solar absorption, though re‐emitted fluorescence mitigates heat generation. In non‐fluorescent CRC coolers, diffuse solar reflectance often shows visible light reflection valleys, indicating solar absorption sites. A trade‐off exists between color vibrancy and cooling performance, depending on the priority of color chroma, saturation, and cooling capacity requirements. Efficient vibrant coloration is achieved with deep, narrow reflection valleys while minimizing solar absorption.^[^ ^66^, ^96^ ^]^ The primary goal in CRC design is to simultaneously achieve vibrant colors, high solar reflectance, and excellent thermal emissivity. Thus, high NIR reflectance and LWIR emittance are preferred, with minimal reflection dips for non‐fluorescent and high PLQY for fluorescent CRC coolers. Finally, we provide key recommendations for achieving vibrant color while minimizing solar absorption for each coloration approach in Table 2.

**Table 1:** A summary of the color and cooling performance of representative CRC coolers.

Categories	Materials	Color	Solar reflectance	LWIR emittance	Outdoor test	Refs.
Photonic crystals	Acrylate/silica colloids	Red/green/blue	≈0.97	≈0.96	≈2 °C below ambient temperature under ≈900 W m^−2^ solar irradiation	[57]
	Silica opals	Red/green/blue	/	/	≈10 °C cooler than black Si plate under ≈900 W m^−2^ solar irradiation	[55]
	TiO_2_/SiO_2_/Si/ SiO_2_/Si/ SiO_2_/ Si/ SiO_2_ stacking structure	Pink	≈0.67	≈0.91	≈19 °C above ambient temperature under over 900 W m^−2^ solar irradiation	[74]
	SiO_2_/SiN/SiO_2_/MaF_2_/SiC/MaF_2_/SiC/Ag stacking structure	Yellow/magenta/cyan	/	/	4.2, 4.6, and 4.9 °C for yellow, magenta, and cyan cooler below ambient temperature under ≈900 W m^−2^ solar irradiation	[92]
	ZnS/TiO_2_/SiO_2_/glass/air gap/Ag/glass multiplayer structure	Yellow	0.958	0.956	7.1 °C below ambient temperature under ≈700 W m^−2^ solar irradiation	[102]
Iridescent periodic interference structures	Tape/PS microsphere array/PDMS/Al	Blue/green/pink	0.10	≈0.96	≈4 °C below ambient temperature under ≈1000 W m^−2^ solar irradiation	[93]
	Polymeric grating (can be stacked on a solar‐reflective sheet)	Iridescent blue/green/red	/	≈0.90	≈2 °C below ambient temperature under ≈1000 W m^−2^ solar irradiation	[34]
Plasmonic resonant structures	Au/Ag nanoparticle/polymer top layer + SiO_2_ nanoparticle/ polymer bottom layer	Light yellow/pink	0.859	0.933	1.9 °C below ambient temperature under ≈880 W m^−2^ solar irradiation	[41]
	Ag nanoantenna secreted in dielectric alternating layers	Gray/purple/blue	0.972/0.97/0.968	≈0.81	/	[45]
Fabry‐Pérot structures	Thermal‐emissive dielectric layers backed an Ag/SiO_2_/Ag cavity	Cyan, magenta, and yellow	/	/	3.9 °C below ambient temperature under ≈1000 W m^−2^ solar irradiation	[46]
	A selective emitter backed an Ag/SiO_x_/Ag cavity	Versatile color by tuning F‐P cavity's geometries	/	≈0.8	/	[101]
Mie resonant structures	Si@SiO_2_ core‐shell nanoparticle/PMMA hybrid coating	Versatile color by tuning Si@SiO_2_ core‐shell nanoparticle's geometries	/	>0.95	Could realize a cooling potential of ≈18 °C compared to the bare Si solar cell	[51]
Solar‐absorptive pigments	Porous P(VdF‐HFP)–based bilayer coating	Black	0.44	0.95	≈14 °C above ambient temperature under ≈960 W m^−2^ solar irradiation	[36]
		Blue	0.40	0.95	≈24 °C above ambient temperature under ≈1000 W m^−2^ solar irradiation	
		Red	0.61	0.95	≈12 °C above ambient temperature under ≈910 W m^−2^ solar irradiation	
		Yellow	0.72	0.95	≈5 °C above ambient temperature under ≈950 W m^−2^ solar irradiation	
	MIR‐transparent color top layer + IR‐reflective layer bottom layer	Blue/red/yellow	/	≈0.15 (≈0.85 of MIR reflectance)	/	[37]
	Colored Infrared Transparent polyethylene textile	Blue/red/yellow	/	≈0.2 (≈0.80 of MIR transmittance)	/	[129]
Fluorescent Pigments	PMMA/SiO_2_@CPB_3_ nanocrystals top layer + ZnO/PET/Ag bottom layer	Green	≈0.81	≈0.95	3.6 °C below ambient temperature under ≈400 W m^−2^ solar irradiation	[56]
	PMMA/SiO_2_ @CPB_x_I_3‐ x_ nanocrystals top layer + ZnO/PET/Ag bottom layer	Red	≈0.78	≈0.95	1.7 °C below ambient temperature under ≈400 W m^−2^ solar irradiation	
	Polyacrylic BaSO_4_/inorganic phosphor top layer + polyacrylic/TiO_2_ bottom layer	Yellow	0.928	0.940	2.5 °C below ambient temperature under ≈1000 W m^−2^ solar irradiation	[130]
		Yellow‐green	0.937	0.930	2.7 °C below ambient temperature under ≈1000 W m^−2^ solar irradiation	
		Green	0.923	0.930	2.2 °C below ambient temperature under ≈1000 W m^−2^ solar irradiation	
		Red	0.908	0.920	0.6 °C below ambient temperature under ≈1000 W m^−2^ solar irradiation	
	CA nanofibers/perovskite quantum dots top layer + CA nanofibrous bottom layer	Green	0.89	≈0.95	5.4 °C below ambient temperature under ≈740 W m^−2^ solar irradiation	[81]
Yellow	0.87	≈0.95	3.4 °C below ambient temperature under ≈740 W m^−2^ solar irradiation
		Red	0.82	≈0.95	2.2 °C below ambient temperature under ≈740 W m^−2^ solar irradiation	
	Polystyrene‐acrylic/ZrO_2_/ inorganic phosphor top layer + Polystyrene‐acrylic/ZrO_2_ bottom layer	Light blue	0.941	≈0.89	4.4 °C below ambient temperature under ≈800 W m^−2^ solar irradiation	[179]
		Blue	0.915	≈0.89	3.5 °C below ambient temperature under ≈800 W m^−2^ solar irradiation	
		Dark blue	0.880	≈0.89	3.0 °C below ambient temperature under ≈800 W m^−2^ solar irradiation	
	Polystyrene‐acrylic/SiO_2_/ inorganic phosphor top layer + Polystyrene‐acrylic/TiO_2_ bottom layer	Red	0.93	≈0.90	0.8 °C below ambient temperature under ≈800 W m^−2^ solar irradiation	[80]
		Yellow	0.94	≈0.90	1.5 °C below ambient temperature under ≈800 W m^−2^ solar irradiation	
		Green	0.936	≈0.90	1.2 °C below ambient temperature under ≈800 W m^−2^ solar irradiation	
CRC with dynamic modulation	Acrylic/hollow glass beads/ thermochromic chameleon microcapsules (CMs) hybrid coating	Blue	92.1, 89.9, and 87.9% for 1, 2, and 5 wt% CMs	≈0.94	≈7.1 °C sub‐ambient cooling under ≈700 W m^−2^ solar irradiation. Additionally, a temperature contrast of 9.5 °C could be achieved when hybridized with CMs.	[25]
		White	95.7%, 95.1%, and 94.7% for 1, 2, and 5 wt% CMs	≈0.94		
	PE/MIR‐transparent pigments/W_x_V_1‐x_O_2_ array/Al/ PET stacking	Blue/yellow/red/white	/	0.25 (cold state); 0.85 (hot state)	/	[61]
	Fiber with thermochromic microparticles/WPU shell and Ag/PU core	Red	/	≈0.95	2.5 °C higher than that of white commercial fabric	[27]
		White	/	≈0.95	2.5 °C lower than that of white commercial fabric	

^*^Data was extracted from corresponding references. “/” denotes that data is not available or not applicable.

**Table 2:** Summary and comparison of two categories of color in CRC materials.

Color categories	Implementation methods	Characteristics and advantages	Recommendations for enhancing color vibrancy and cooling performance	Limitations	Refs.
Photonic Structure‐based	Photonic crystals	Selective reflection of the desired color via deliciated photonic design; thermal emissivity can be engineered simultaneously	Optimizing the structure and constituent materials such as periodicities and featured sizes is needed to reflect vibrant colors and ensure high NIR reflectivity and MIR emissivity.	High cost; Limited scalability	[55, 57, 74, 92, 102]
	Fabry‐Pérot resonant structures	Selectively reflect/transmit the desired color; feasible assembly with a thermal emitter	By controlling the cavity and mirror thickness, a reflection dip at a specific wavelength can be achieved for the desired vibrant color and minimal solar absorption.	High cost; Limited scalability	[46, 101]
	Mie resonant structures	Selective reflection of the desired color; potential scalability	By fine tuning the size and shape of the Mie resonant structure, vibrant colors can be reflected in a type of narrowband reflection valley with minimal solar absorption.	Precise control of photonic structures’ geometry	[51]
	Plasmon resonant structures	Visible absorption for the desired coloration; potential scalability	The geometric size of plasmonic structures such as Ag or TiO_2_@Ag core‐shell nanoparticles is delicately controlled to achieve narrowband visible absorption for minimal solar heat and vibrant colors.	Relatively high production cost; precise control of photonic structures’ geometry	[44, 45, 96]
	Iridescent periodic interference structures	Colors are reflected in different views by interference; scalability such as roll‐to‐roll fabrication	Optically lossless materials with high thermal emission such as transparent organic polymers and SiO_2_ are usually configured into the interference structure atop a reflective bottom for vivid color and minimal solar absorption	Iridescent structural color may not meet practical aesthetic requirements.	[34, 35, 93, 107]
Optical material‐based	Solar absorptive pigments	Versatile color options; low‐cost; high scalability	The use of low concentration of pigments is positive for minimal solar absorption but could only produce light colors.	Wideband solar absorption	[36, 37, 129]
	Fluorescent pigments	Absorbed photon conversion and fluorescence reemission for reducing solar absorption and coloration; high scalability	Pigments with high PLQY and the appropriate wavelength shift are preferred for producing vibrant colors and compensating solar absorption.	Relatively high material cost	[23, 56, 80, 81, 130, 179]

**Figure 13:**
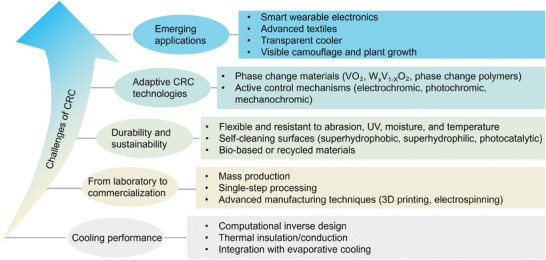
Challenges in the development of colored radiative cooling.

The integration of advanced photonic approaches into colored radiative cooling represents a promising frontier that bridges aesthetics, functionality, and sustainability. While significant progress has been made in recent years, several challenges remain to be addressed for this field to reach its full potential. To better illustrate the challenges and solution strategies in the development of colored radiative cooling materials, we have included a summary in **Figure** **13**. This figure offers a clear visualization of the proposed strategy for overcoming the identified challenges, enabling readers to gain a deeper understanding of the research landscape and potential directions for future work.

1. There remains significant potential for enhancing the radiative cooling performance of CRC materials, particularly in optimizing spectral properties such as the color chroma, solar reflectance, and infrared emissivity. A promising direction for advancement involves harnessing computational inverse design approaches,^[^ ^180^, ^181^, ^182^ ^]^ where machine learning significantly accelerates and optimizes the design process. These methods have proven effective in the development of multilayer film‐based radiative coolers,^[^ ^101^, ^183^ ^]^ and nanoparticle‐embedded structures.^[^ ^184^ ^]^ Implementing such techniques could open new possibilities for the precise and efficient design of colored radiative coolers, further improving their cooling efficiency. To further optimize cooling performance, thermal insulating materials such as aerogels are employed to minimize non‐radiative heat exchange, enabling efficient sub‐ambient radiative cooling.^[^ ^185^, ^186^ ^]^ Conversely, thermally conductive materials like boron nitride are utilized to enhance non‐radiative heat transfer, facilitating effective above‐ambient radiative cooling.^[^ ^18^, ^187^ ^]^ Gradient‐structured porous polymers also provide asymmetric thermal insulation, blocking outdoor heat while promoting indoor heat dissipation, making them ideal for thermal rectification in enclosed spaces.^[^ ^188^ ^]^ Combining these materials with CRC coatings can significantly improve cooling efficiency while preserving aesthetic appeal. Additionally, integrating CRC with evaporative cooling through hydrogels^[^ ^189^ ^]^ or hygroscopic salts^[^ ^190^ ^]^ can address challenges posed by humid environments.
2. A significant challenge in the development of CRC technologies is transitioning from laboratory prototypes to commercially viable products. For widespread adoption, CRC materials must be designed for mass production using conventional industrial techniques such as brushing, rolling, and spraying.^[^ ^191^ ^]^ Although roll‐to‐roll fabrication holds great promise for scalable production,^[^ ^35^ ^]^ several factors remain to be addressed, including material costs, the toxicity of raw materials, and the environmental impact of the manufacturing process. To ensure economic feasibility, it is crucial to develop straightforward, scalable fabrication methods, such as single‐step processes, to reduce complexity and production costs. Furthermore, CRC coatings must be mechanically robust, easy to install, and compatible with various surface types, such as building exteriors, textiles, and flexible substrates. Advanced fabrication methods, like 3D printing^[^ ^192^ ^]^ and electrospinning techniques,^[^ ^162^ ^]^ could be used to ensure mechanical flexibility to various substrates.
3. The advancement of self‐adaptive CRC technologies represents a critical step towards intelligent thermal management systems. Conventional thermochromic materials primarily focus on modulating visible light absorption, but they neglect the regulation of NIR and MIR wavelengths. This limitation undermines overall cooling efficiency and may affect visual aesthetics.^[^ ^25^ ^]^ By incorporating various phase‐changing materials like VO_2_ or W_x_V_1‐x_O_2_ into CRC design, it is possible to achieve modulation of optical properties, including NIR reflection and thermal emittance, without altering the color.^[^ ^76^, ^113^ ^]^ Future advancements in phase‐change polymers for multi‐band adaptive CRC systems are expected, as their flexibility and compatibility with scalable, cost‐effective manufacturing offer promising opportunities to expand the applications of advanced thermal regulation materials. In addition to PCMs, it is essential to explore alternative active control mechanisms such as electrochromic, photochromic, and mechanochromic technologies, to achieve dynamic control over radiative cooling.^[^ ^193^ ^]^ These advanced technologies can significantly enhance the energy efficiency of CRC systems by facilitating real‐time thermal regulation in response to environmental changes. For example, electro‐driven dynamic radiative materials with switchable heating, white cooling, and multicolored cooling modes exhibit outstanding energy modulation, achieving power density variations up to 659 W m⁻^2^ and temperature shifts of as much as 11 °C.^[^ ^194^ ^]^
4. Moreover, it is crucial to prioritize durability and sustainability in CRC technology. Challenges such as cracking, peeling, and spalling of CRC coatings frequently arise, affecting both their aesthetic appearance and cooling performance. The mechanical properties of CRC materials such as toughness, hardness, and elastic modulus have to be optimized to meet the specific requirements of different applications. For instance, coatings for wearable electronics need to be flexible and abrasion‐resistant, while coatings for building exteriors must withstand UV exposure, humidity, and temperature fluctuations. A crucial aspect of durability is the self‐cleaning capability of CRC coatings, which helps maintain their optical performance and reduces the need for frequent maintenance. Enhancing self‐cleaning performance can be achieved by designing superhydrophobic or superhydrophilic surfaces.^[^ ^195^, ^196^, ^197^ ^]^ Superhydrophobic surfaces, characterized by water contact angles greater than 150°, can be achieved by introducing micro‐ or nanoscale features that promote water repellency. For example, micropillar arrays can increase the water contact angle, allowing droplets to roll off easily and carry away dirt. Conversely, superhydrophilic surfaces, with contact angles below 5°, can be realized by structures like nanoholes or nanopillars, which enable water to spread uniformly and rinse away contaminants.^[^ ^197^ ^]^ Another promising approach to enhancing self‐cleaning performance is incorporating photocatalytic properties to degrade organic contaminants.^[^ ^198^, ^199^ ^]^ This can be achieved by integrating photocatalytic materials such as TiO₂^[^ ^200^ ^]^ or ZnO,^[^ ^201^ ^]^ which generates reactive oxygen species under UV or visible light exposure. These reactive species effectively break down organic pollutants on the surface, maintaining long‐term cleanliness and reducing maintenance requirements. Future research should explore the use of biodegradable, recyclable, or renewable materials in photonic designs to minimize waste and ensure long‐term sustainability. Exploring affordable and sustainable alternatives to high‐cost materials, such as bio‐based options like cellulose‐based films^[^ ^105^ ^]^ and photonic pigments,^[^ ^202^ ^]^ or recyclable materials like recycled polystyrene foam, printer paper,^[^ ^203^ ^]^ and recycled acrylic sheets,^[^ ^204^ ^]^ presents a promising solution.
5. Furthermore, several new factors need to be considered when utilizing CRC technology, particularly in conjunction with wearable electronics^[^ ^18^ ^]^ and advanced textiles.^[^ ^163^ ^]^ Electronic devices often incorporate cutting‐edge functionalities like sensing,^[^ ^205^, ^206^ ^]^ computing,^[^ ^207^, ^208^ ^]^ and self‐powering.^[^ ^209^, ^210^ ^]^ By ensuring compatibility of CRC technology with these features, advanced thermal management of wearable electronics and textiles can be achieved without compromising performance. Additionally, visually transparent thermal management photonic techniques have potential applications on pre‐colored surfaces. For example, a visually transparent cooler applied to the exterior of a colored wall can exhibit low transmittance in the UV and NIR regions while simultaneously demonstrating high emissivity in the LWIR spectrum.^[^ ^68^ ^]^ This photonic cooler, with its high visible spectrum transmittance in the visible spectrum, can effectively provide cooling without altering the object's color appearance. Moreover, another two specific application scenarios for CRC technology include its integration with camouflage^[^ ^28^, ^29^ ^]^ and thermal light management of plant growth.^[^ ^30^, ^31^ ^]^ These applications broaden the scope of CRC technology to military uses and enhance vegetation production.

In conclusion, compared with traditional white RC materials, CRC materials prove more appealing and harbor significant potential for decoration, light pollution mitigation, and multifunctionality development. By addressing challenges related to scalability, durability, and sustainability, and by exploring emerging applications in buildings, wearable electronics, textiles, and other applications, CRC technologies hold the potential to contribute significantly to global energy conservation and sustainability goals. Continued efforts in material innovation, process optimization, and interdisciplinary research will be essential to fully realize the transformative potential of CRC technologies across diverse applications.

## Conflict of Interest

The authors declare no conflict of interest.

## Author Contributions

T.W. and Y.L. contributed equally to this work. Y.D. and X.B.Y. supervised the work and reviewed the manuscript. D.Y.L. and J.G.D. supervised the work, wrote, edited, and reviewed the manuscript, and investigated the project.
